# Sheltered in Stromal Tissue Cells, *Trypanosoma cruzi* Orchestrates Inflammatory Neovascularization via Activation of the Mast Cell Chymase Pathway

**DOI:** 10.3390/pathogens11020187

**Published:** 2022-01-29

**Authors:** Lucas Vellasco, Erik Svensjö, Carlos Alberto Bulant, Pablo Javier Blanco, Fábio Nogueira, Gilberto Domont, Natália Pinto de Almeida, Clarissa Rodrigues Nascimento, Danielle Silva-dos-Santos, Carla Eponina Carvalho-Pinto, Emiliano Horácio Medei, Igor C. Almeida, Julio Scharfstein

**Affiliations:** 1 Department of Immunobiology, Institute of Biophysics Carlos Chagas Filho, Federal University of Rio de Janeiro, Rio de Janeiro 21941-902, Brazil; lucasvellasco@hotmail.com (L.V.); erik.svensjo@gmail.com (E.S.); ufrjclarissa@gmail.com (C.R.N.); danielle_santos@hotmail.com (D.S.-d.-S.); emedei70@gmail.com (E.H.M.); 2 Department of Mathematical and Computational Methods, National Laboratory for Scientific Computing, Petrópolis 25651-075, Brazil; carlos.alberto.bulant@gmail.com (C.A.B.); pablo.j.blanco@gmail.com (P.J.B.); 3 Department of Biochemistry, Institute of Chemistry, Federal University of Rio de Janeiro, Rio de Janeiro 21941-909, Brazil; fabiocsn@gmail.com (F.N.); gilbertodomont@gmail.com (G.D.); natalia1almeida@hotmail.com (N.P.d.A.); 4 Department of Immunobiology, Institute of Biology, Federal Fluminense University, Niterói 24210-201, Brazil; carlaeponina@gmail.com; 5 Department of Biological Sciences, Border Biomedical Research Center, University of Texas at El Paso, El Paso, TX 79968, USA; icalmeida@utep.edu

**Keywords:** *Trypanosoma cruzi*, angiogenesis, inflammation, mast cell, chymase

## Abstract

Microangiopathy may worsen the clinical outcome of Chagas disease. Given the obstacles to investigating the dynamics of inflammation and angiogenesis in heart tissues parasitized by *Trypanosoma cruzi*, here we used intravital microscopy (IVM) to investigate microcirculatory alterations in the hamster cheek pouch (HCP) infected by green fluorescent protein-expressing *T. cruzi* (GFP-*T. cruzi*). IVM performed 3 days post-infection (3 dpi) consistently showed increased baseline levels of plasma extravasation. Illustrating the reciprocal benefits that microvascular leakage brings to the host-parasite relationship, these findings suggest that intracellular amastigotes, acting from inside out, stimulate angiogenesis while enhancing the delivery of plasma-borne nutrients and prosurvival factors to the infection foci. Using a computer-based analysis of images (3 dpi), we found that proangiogenic indexes were positively correlated with transcriptional levels of proinflammatory cytokines (pro-IL1β and IFN-γ). Intracellular GFP-parasites were targeted by delaying for 24 h the oral administration of the trypanocidal drug benznidazole. A classification algorithm showed that benznidazole (>24 h) blunted angiogenesis (7 dpi) in the HCP. Unbiased proteomics (3 dpi) combined to pharmacological targeting of chymase with two inhibitors (chymostatin and TY-51469) linked *T. cruzi*-induced neovascularization (7 dpi) to the proangiogenic activity of chymase, a serine protease stored in secretory granules from mast cells.

## 1. Introduction

Chagas disease (CD), the chronic infection caused by the parasitic protozoan *Trypanosoma cruzi*, afflicts approximately 6–8 million people in Central and South America [1,2]. Having diverged from *T. brucei* following the breakup of Gondwanaland ~100 millions of years ago [3], the South American trypanosomatid species evolved the ability to invade and develop intracellularly, in obligate manner. It is currently subclassified into seven discrete typing units (DTU; TcI-TcVI and Tc-bat) [4,5]. Subjected to selective pressure by hundreds of triatomine insects and wild mammals in the sylvatic environment, *T. cruzi* genomic plasticity was shaped by varying the content and sequence diversity of multicopy gene families, including those coding for parasite virulence factors, such as *trans*-sialidase (TS), mucin-associate surface protein (MASP), mucin, and GP63 protein families [6].

Natural infection by *T. cruzi* usually starts following a blood meal by infected triatomine insects. Once released in fecal fluids, infective metacyclic trypomastigote forms invade susceptible cells in the lacerated skin tissues, or exposed oral/ocular mucosa, and/or enter the bloodstream. Outbreaks of oral infection have been reported due to accidental ingestion of contaminated fruit juices and food stocks [7]. Although tissue tropism and host-cell preference of *T. cruzi* vary considerably between different strains and genotypes [1,5], in vitro studies of trypomastigote interaction with cardiovascular cells demonstrated that parasite invasion is potentiated by interdependent signaling of G-protein-coupled bradykinin and endothelin receptors [8,9,10,11,12]. After exiting the parasitophorous vacuole, the flagellated trypomastigotes transform into ova-shaped amastigotes, which then undergo multiple cycles of binary division before transforming again into virulent trypomastigotes. Commonly lasting 6–7 days in cell culture, the asynchronous [13] process of intracellular parasite development is linked to upregulated expression of surface antigens encoded by polymorphic multigene families, such as mucin, TS, MASP, and GP63—all of which anchored to the plasma membrane by glycosylphosphatidylinositol (GPI) [14,15,16]. Upon host cell death, the injured tissues are simultaneously exposed to a myriad of alert cues originating from necrotic cells and to proinflammatory trypomastigotes. Orchestrated by Toll-like receptor 2 (TLR2)-expressing innate sentinel cells, such as macrophages and dendritic cells, innate immunity is robustly activated by trypomastigote mucin-derived GPIs (tGPIs) [17], a potent TLR2 agonist [18] exported in extracellular vesicles shed by the trypomastigotes [19]. Immunological studies showed that tGPIs induce neutrophil-dependent extravasation of plasma via activation of the TLR2/CXCR2 pathway [20]. Further downstream, trypomastigote-derived cruzipain proteolytically releases bradykinin, a short-lived peptide that, acting jointly with endothelin-1, fuels heart parasitism [21,22]. Reciprocally, bradykinin stimulates the intralymphoid development of IFN-γ-producing T cells [23], via crosstalk between G-protein-coupled bradykinin (B2) [24] and C5a (C5aR1) receptors [25].

Acute Chagas disease is associated to high blood parasitemia and systemic inflammation. Lasting weeks, the acute manifestations usually subside upon mobilization of the adaptive arm of immunity [26]. Benefiting from the combined protective action of lytic anti-α-Gal antibodies [27,28,29] and type-1 effector T cells [30], the patients develop an asymptomatic infection that may last years. Exploiting fluctuations in immune regulatory networks [31], *T. cruzi* succeeds in maintaining a low-grade infection in immunoprivileged tissues [32,33,34]. For reasons that are still unclear, about 20–30% of the patients develop a progressive form of chronic Chagas disease cardiomyopathy (CCC), which may be fatal due to cardiac arrhythmias, congestive failure, stroke, or thromboembolism [1,2].

Whether involving persistent myocardial infection [35] or transient (“hit-and-run”) cycles of intracardiac parasitism [36], the concept that low-grade myocardial parasitism is a prerequisite for the development of chronic myocarditis is now well-accepted. Systematic analyses of intracardiac T cell infiltrates in murine models of CCC linked the severity of the myocarditis to the unbalanced recruitment of effector T cells that are either protective (IFN-γ-producing CD8^+^) or detrimental (perforin-producing CD8^+^ T cells) [37].

Advocates of the vascular theory [38] proposed long ago that CCC is worsened by convergence of T-cell-dependent immunopathology and infection-associated microangiopathy [39]. More recently, clinical studies showed a hypercoagulable phenotype in a cohort of Bolivian patients who immigrated to Spain [40]. It is unclear whether microthrombi are transiently formed in the myocardium, either reflecting endothelial injury caused by infection [1,7,9,11,12], or by proinflammatory cytokines secreted by parasite-specific effector T lymphocytes [41]. In a pioneer study, Tanowitz and co-workers [42] provided evidence that heart fibrosis was worsened by endothelin-1, a prooxidative polypeptide whose expression is upregulated in parasitized cardiomyocytes.

Strategically localized in perivascular regions, MCs from connective tissue and mucosa differ in quantity and repertoire of enzymes and mediators stored in secretory granules [43]. Studies of the pathogenesis of human cardiomyopathies [44] and viral myocarditis in animal models [45] linked MC-derived serine proteases (e.g., tryptase and chymase) to metalloproteinase-dependent tissue remodeling. More recently, it was demonstrated that human and mouse chymase (MCP-4) convert big endothelin 1 (big ET-1) into the potent vasopressor ET-1 [46]. Further highlighting the potential role of cardiac mast cells in heart inflammatory diseases, it has been reported that chymase generates angiotensin II in human and animal hearts independently of the angiotensin-converting enzyme (ACE) [47,48,49]. Interestingly, hamsters and dogs develop a dilated chronic cardiomyopathy [50,51] that closely resembles human CCC.

Angiogenesis was the object of limited studies in mice acutely challenged systemically by two different *T. cruzi* strains [52,53]. Using IVM, we recently developed computerized algorithms [54] that measure and discriminate proinflammatory and proangiogenic responses in hamster cheek pouch tissues parasitized by Dm28c *T. cruzi.* Here, we provide evidence that these parasites, acting inside out as inducers of plasma leakage, coordinate angiogenesis via activation of the MC-chymase pathway.

## 2. Results

### 2.1. Microvascular Profiles in HCP Tissues Infected by GFP-T. cruzi

**Figure 1:**
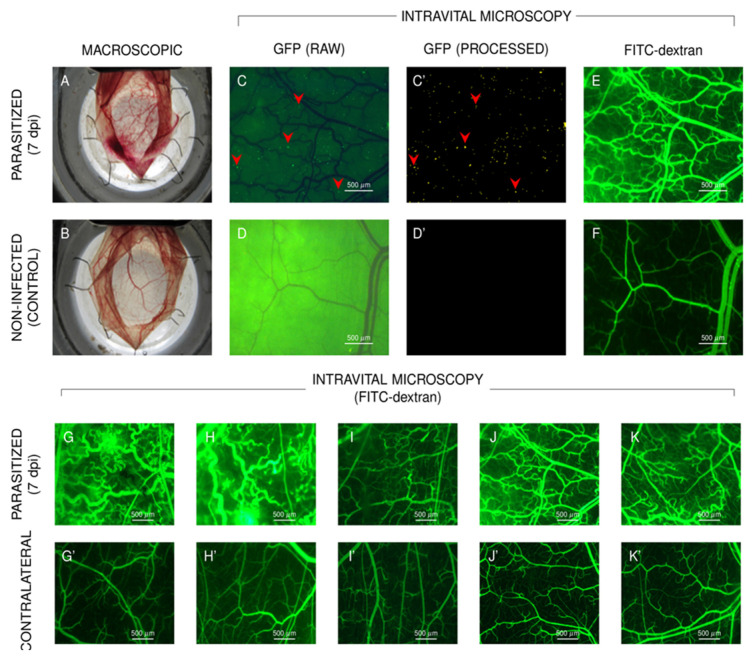
**Microvascular profile of *T. cruzi*-infected cheek pouches.** Macroscopic view of (**A**) HCP inoculated 7 days earlier with Dm28c WT-TCTs versus (**B**) normal HCP. (**C**) IVM image of HCP inoculated 7 days earlier with GFP-TCTs. (**C’**) Visible GFP aggregates (300–1400 patches per 5 mm^2^). (**D**,**D’**) IVM with absence of GFP patches in noninfected HCP (controls). (**E**) IVM image of parasitized HCP (7 dpi) recorded 90 min after i.v. injection of 150-kDa FITC-dextran. (**F**) FITC-dextran profile of microvasculature of noninfected controls. (**G**–**K**,**G’**–**K’**) FITC-dextran tracing of the cheek pouch microcirculation of five hamsters. Upper panel (**G** > **K**): FITC-dextran tracing of the left pouch inoculated 7 days earlier with GFP-TCT. Lower panel (**G’** > **K’**): FITC-dextran tracing of microvessels from the contralateral pouches (noninfected) of the same hamsters. Statistical comparison of measurements of angiogenic indexes in left (infected) and right HCPs (controls) are shown in Figure S1.

Extending the breadth of previous study [54], in the current work we sought to systematically investigate the dynamics of inflammatory neovascularization in HCP tissues parasitized by *T. cruzi*. Briefly, after externalizing the HCP, a PBS suspension of wild-type (WT) Dm28c tissue culture-derived trypomastigotes (TCTs) was delicately inoculated in the hyaluronic-rich layer covering the vascularized part of the left HCP. Seven days later, the macroscopic visualization of the externalized HCP revealed overt changes in the microvascular beds, including punctual hemorrhagic lesions (Figure 1A). In contrast, there was no macroscopic sign of lesions in the HCPs from normal hamsters (Figure 1B), nor in the contralateral pouch inoculated 7 days earlier with PBS (Figure S1A,B). We then challenged the HCPs with the same dose of GFP-TCTs (Dm28c), and found that the genetically altered parasites also induced lesions (7 dpi) that were discerned macroscopically. Although we did not detect intracellular nests of GFP-*T. cruzi* using the optical lenses (low resolution) routinely employed in IVM, we were surprised to find a large number of patchy deposits (around 300 per 5 mm^2^) of parasite-derived GFP dispersed (7 dpi) in the tissue stroma (Figure 1C,C’). As predicted, these GFP clusters were not observed (7dpi) in the contralateral HCPs (7 dpi) (Figure 1D,D’). Next, we injected (i.v.) the macromolecular tracer FITC-dextran (150 kDa) in the anesthetized hamsters, and asked whether infection by GFP-*T. cruzi* altered microvascular homeostasis. IVM images captured at 7 dpi indicated that the density of microvessels labelled by FITC-dextran was robustly increased in parasitized tissues (Figure 1E), contrasting to the microvessel distribution in control (noninfected) HCPs (Figure 1F). In a separate experiment involving seven hamsters infected by GFP-TCTs, we compared the microvessel distribution of FITC-dextran in the left HCPs (7 dpi; Figure 1G,K; for simplicity, the lower panel only show images of five animals) compared to representative images of contralateral pouches (noninfected controls; Figure 1G’,K’). Next, the same cohort of hamsters were studied by IVM using the digital methodology developed by Bulant et al. [54]. We found that most proinflammatory and proangiogenic geometric indexes of the infected HCPs (7 dpi) were increased in relation to the values of the contralateral (noninfected) pouches (Figure S1).

In a follow-up experiment, the left HCP was challenged with increasing inocula of GFP-TCTs (10^4^,10^5^, 5 × 10^5^, and 10^6^). Measurements of microvascular parameters (7 dpi) revealed a positive correlation between the parasite inocula and two values of two indexes: (i) relative fluorescence units (RFU, *n* = 5, *r* = 0.833, *p* = 0.0796) and (ii) total vascular length (TVL, *n* = 5, *r* = 0.917, *p* = 0.0284) (Figure S2A,B). It is noteworthy that control studies showed that HCPs (left) injected seven days earlier with PBS (*n* = 5) were not different from those of a larger sample of normal and noninjected HCPs (*n* = 40) (Figure S3).

### 2.2. Asynchronous Intracellular Development of T. cruzi Influences the Dynamics of Inflammatory Neovascularization

**Figure 2:**
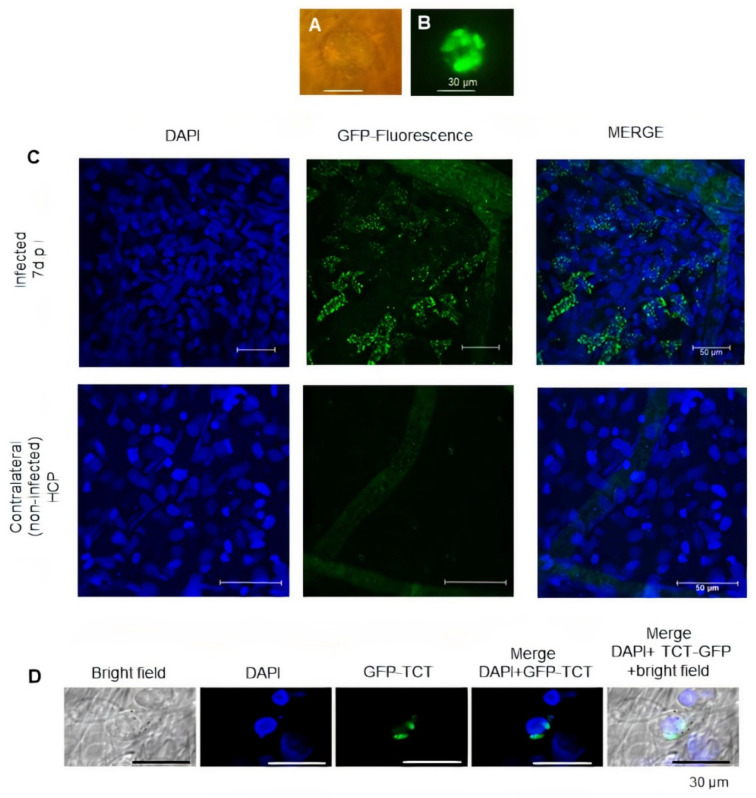
**Detection of intracellular GFP-*T. cruzi* in hamster cheek pouch tissues**. (**A**) IVM image of the infected HCP (7 dpi) under transparent light. (**B**) Same tissue, showing the GFP fluorescence inside enlarged cellular structures. (**C**) Confocal microscopy showing multiple intracellular forms of GFP-*T. cruzi* in the left HCP (7 dpi, upper panel, magnification 400×, scale bar size 50 µm). Controls (bottom panels, magnification 630×, scale bar size 50 µm) represent images of fixed tissues from the contralateral (noninfected) pouch of the same hamster; a faint FITC-dextran tracing of microvessels is observed in these controls (middle and right panels). (**D**) Deconvoluted microscopy showing representative image of an infected cell harboring a few GFP-T. cruzi (7 dpi); scale bar size 30 µm; magnification 630×. Data representative of two infected hamsters.

As mentioned earlier in this section, the power of the optical lenses routinely used in IVM did not allow us to visualize intracellular clusters of GFP-*T. cruzi*. However, using a water immersion lens (20×), it was observed that the HCP tissues (7 dpi) contained a large number of enlarged spherical fluorescent structures (~50 µm) originating from GFP-expressing parasites (Figure 2A,B). We then fixed control and the infected tissues and analyzed these images by confocal microscopy. As predicted, we found extensive numbers of host cells harboring GFP-*T. cruzi* in the HCPs (Figure 2C). It is noteworthy that using 3D-deconvolution microscopy at 7 dpi, we could discern the presence of stromal cells harboring just a few GFP-labeled parasites (Figure 2D). Since host-cell invasion and parasite outgrowth in culture systems is an asynchronous process, this image (7 dpi) may represent secondary infection forged by trypomastigotes that were precociously released (<7 dpi) from ruptured/dead stromal cells. Alternatively, this image may represent an innate sentinel cell type that, being previously infected (<7 dpi), has succeeded in limiting the extent of intracellular amastigote outgrowth. Figure 2 had a title included in the image. We removed the title to be consistent with the figures in the text and also meet the requirements for figures identification.

### 2.3. Tracking the Dissemination of Luc-Expressing T. cruzi following HCP Colonization

**Figure 3:**
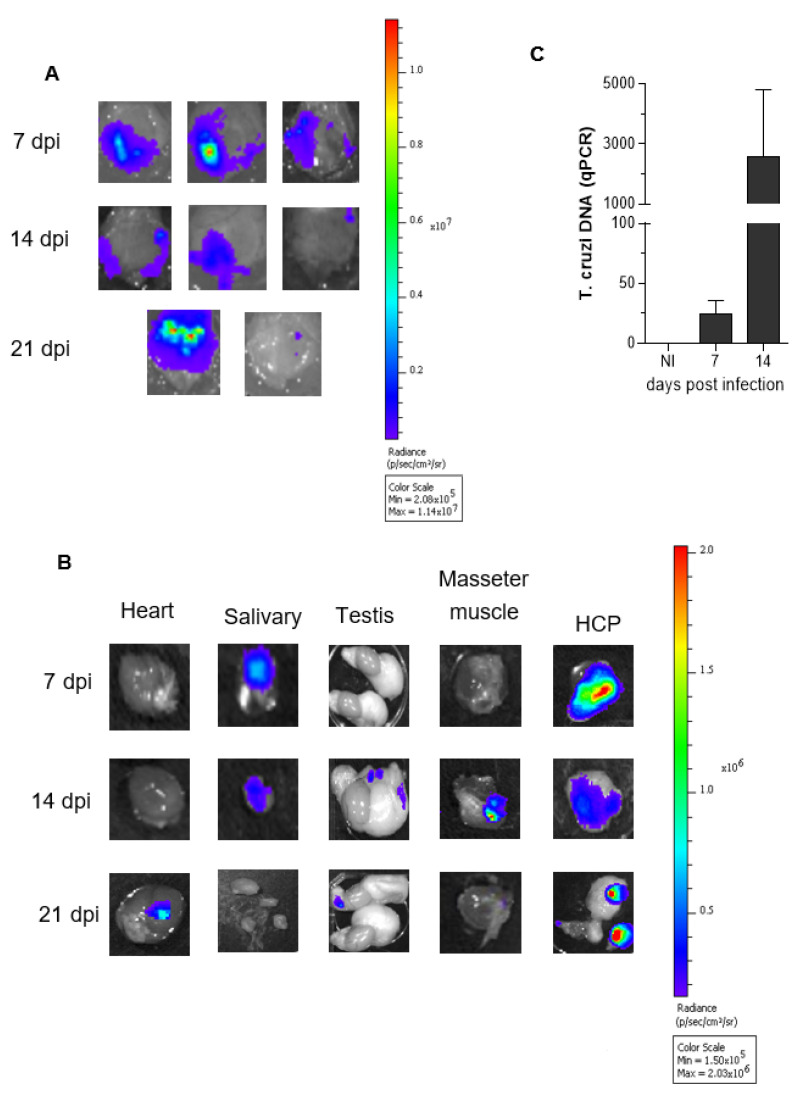
**Tracking the migration of luc-*T. cruzi* after HCP colonization**. (**A**), Bioluminescence images obtained 7 days after inoculating the left HCP with a 1:1 mixture of Dm28c luc-TCTs and GFP-TCTs). At different time-points (7, 14, and 21 dpi), luciferin was injected (i.v.) to detect bioluminescence emitted by luc-*T. cruzi*. (**A**) Images of the left cheek pouch (8 outbred hamsters) show that bioluminescence signals vary as the infection progresses. (**B**) Images show variable bioluminescence (7, 14, and 21 dpi) in the HCP fat tissues, salivary lymph nodes, testis, masseter muscle, and heart tissues. Scales represent the intensity of bioluminescence in radiance (p/s/cm^2^/sr). (**C**) *T. cruzi* DNA in heart tissues was measured by qPCR on 0, 7 dpi (*n* = 2), 14 dpi (*n* = 2).

Considering that the first intracellular cycle of *T. cruzi* development is concluded with host cell death and release of the intracellular trypomastigotes, we speculated that the extracellular parasites may exploit the dense network of capillary vessels to spread the infection systemically, reminiscent of the role of angiogenesis in tumor metastasis. As a start point in the investigation of this challenging hypothesis, we studied the dynamics of tissue migration of luc-*T. cruzi*. The experiment design involved inoculation of equal proportions of DM28c luc-TCTs and GFP-TCTs (total 10^6^ parasites) in the left HCPs of naïve hamsters. For internal controls, we performed IVM in a separate group of animals to make sure that the parasitized HCPs (7 dpi) (i) exhibited a high density of GFP deposits as an indicator of robust tissue parasitism, and (ii) displayed a dense network of FITC-labelled capillaries. Having confirmed that both premises were met, we next sought to monitor the tissue-migration profile of luc-*T. cruzi* by injecting the hamsters with luciferin (i.v.) at 7, 14, and 21 dpi. The extent of parasite burden in the HCPs varied considerably from one genetically outbred animal to another; in most, the bioluminescence decayed after 7 dpi, however, in a few hamsters, the signals persisted up to 21 dpi (Figure 3A). Interestingly, we also spotted luc-parasites in fatty tissues that were physically proximal to the primary site of infection (Figure 3A; 7 dpi (*n* = 3), 14 dpi (*n* = 3), and 21 dpi (*n* = 2) (Figure 3B, right side of the panel). The migration of luc-*T. cruzi* to more distant tissues varied within the hamster cohort, sometimes involving salivary lymph nodes (7 and 14 dpi), testis (14 and 21 dpi), and heart (21 dpi) (Figure 3B). To confirm that parasites had colonized the cardiac tissues of the latter subset of animals, we next measured *T. cruzi* DNA by qPCR. Our results showed low levels of *T. cruzi* DNA at 7 dpi (*n* = 2). However, at 14 dpi, the qPCR data revealed that the intracardiac load of parasites was sharply increased (Figure 3C).

### 2.4. Angiogenesis Depends on Intracellular Parasitism

**Figure 4:**
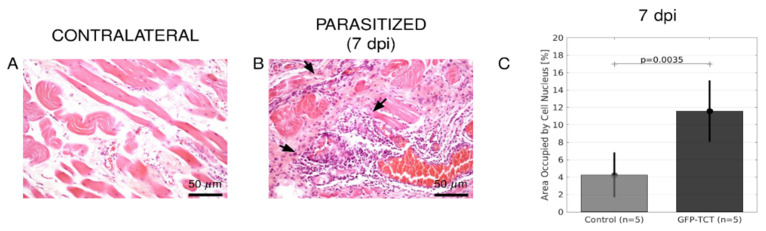
**Histopathological analyses of the parasitized HCP.** Hamsters were inoculated with GFP-TCT in the left cheek pouch. Seven days later, the infected HCP tissues were fixed and stained by H&E to characterize the histopathological features (**B**) as compared to noninfected controls ((**A**), noninfected/contralateral HCP). (**C**) representing area occupied by cell nucleus (images in 20× magnification, 50-µm scale bar) shows increased leukocyte infiltration in *T. cruzi*-infected HCP. Statistical analysis was performed by unilateral paired Student’s *t*-test).

To further characterize the pathological features associated to tissue parasitism, we performed H&E staining (7 dpi) and found that leukocyte infiltration was 2.3-fold higher in the parasitized/inflamed HCPs as compared to controls (contralateral tissues) (Figure 4, *p* = 0.0035). Not surprisingly, we found conspicuous injuries in HCPs at this time-point (7 dpi). As mentioned later in this section, H&E failed to detect leukocyte infiltration at 3 dpi, strongly suggesting that tissue injuries are caused by extensive host cell death and exposure of injured tissues to proinflammatory trypomastigotes at the end of the asynchronous intracellular cycle of parasite development (Figure 4).

**Figure 5:**
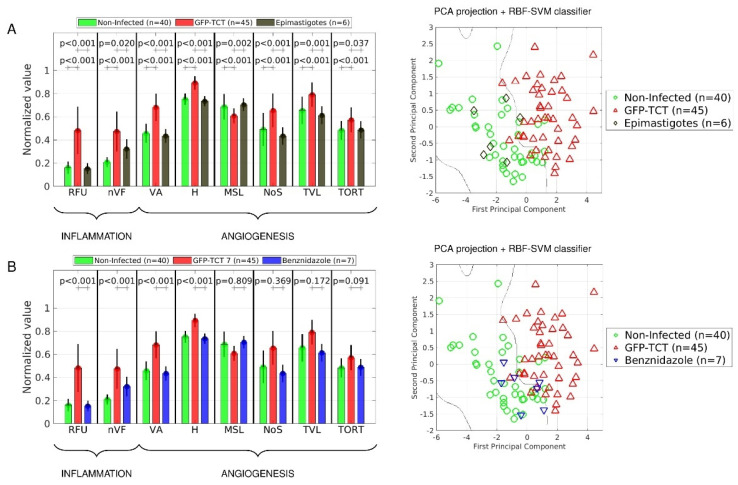
**Inflammatory neovascularization depends on *T. cruzi* infectivity.** (**A**) **Left panel**: Comparison of means ± SD of different inflammatory and angiogenic parameters (RFU, nVF, VA, NoS, TVL, H, MSL, and TORT) of control (noninfected) HCPs (green, *n* = 40) versus HCPs inoculated seven days earlier with 10^6^GFP-TCTs (red, *n* = 45) or 10^6^ of GFP-Epis (dark yellow, *n* = 6). **Right panel**: The complexity of the microvascular architecture of the HCP was analyzed using the classifier algorithm PCA-RBF-SVM. The decision boundary that separates areas of the microvascular architecture of normal HCP (green diamonds) versus parasitized (7 dpi) tissues (red diamonds). The PCA classification of GFP-Epis HCPs (dark yellow diamonds) was performed in blind manner. (**B**) **Left panel:** Comparison of means ± SD of different inflammatory and angiogenic parameters (RFU, nVF, VA, NoS, TVL, H, MSL, and TORT) in infected versus Bz-treated infected hamsters. Treatment (oral) with the trypanocidal drug (100 mg/kg) started 24 h after inoculating the HCPs (left) with GFP-TCTs and was continued for 4 days (blue, *n* = 7). **Right panel**: Comparison of the complexity of the microvascular architecture of BZ-treated HCP with reference boundaries defined by the PCA classifier as described above. Bz (>24 h)-treated HCP (dark blue diamonds, *n* = 7). Statistical analysis was performed with a two-tailed Mann–Whitney nonparametric test. *p*-values are indicated for the actual comparison between groups; GFP-TCTs (7 dpi) versus noninfected HCP, versus GFP-Epis, or versus GFP-TCTs (7 dpi) treated with Bz (>24 h).

We next asked whether *T. cruzi* infectivity was a prerequisite for induction of angiogenesis. To address this question, we injected equal inocula of Dm28c GFP-TCTs or noninfective Dm28c epimastigotes (GFP-Epis) in the HCPs of separate groups of hamsters, and examined the tissue distribution of GFP deposits at 7 dpi. Unlike the dense/scattered distribution of GFP deposits observed in tissues (7 dpi) challenged with GFP-TCTs (Figure 1G,K), we did not find GFP-fluorescent aggregates in HCPs inoculated with GFP-Epis. We then injected FITC-dextran (i.v.) in both groups, and used IVM combined with our digital methods to measure indexes of (i) inflammation (RFU and nVF) and (ii) angiogenesis (VA, H, MSL, NoS, TVL, and TORT). As predicted, RFU and nVF were not increased in HCPs injected 7 d earlier with GFP-Epis (Figure 5A). Likewise, measurements of six proangiogenic indexes (including MSL, whose values are inversely related to the extent of microvessel sprouting) of HCPs, inoculated seven days earlier with GFP-Epis, displayed reduced values as compared to tissues inoculated with GFP-TCTs (Figure 5A, left panel). We then used a classifier algorithm (see Material and Methods) to compare the global complexity of the microvasculature of both cohorts at the same timepoint. Our results (Figure 5A, right panel) showed that 83.3% of the microvascular profiles of tissues challenged by GFP-Epis were classified within the area of noninfected controls. Overall, these data show that the extent of angiogenesis increases as a function of *T. cruzi* virulence/pathogenicity.

To investigate the role of intracellular parasitism in inflammatory neovascularization, we next sought to measure microvascular responses in infected HCPs (7 dpi) that were continuously treated with benznidazole (Bz), a trypanocidal drug commonly used in chemotherapy of CD [55]. Importantly, the onset of drug administration (oral) was delayed for over 24 h (Bz > 24 h) to spare the inoculated GFP-TCTs from drug toxicity while providing these infective forms with comfortable time to probe and invade susceptible stromal cells. To make sure that the trypanocidal drug would preferentially target the intracellular stages of *T. cruzi*, we continued the oral treatment with Bz for another 5–6 days. Importantly, drug targeting of *T. cruzi* with this potent trypanocidal drug prevented the formation of GFP-patches in the HCPs. Having confirmed that tissue parasitism was drastically reduced by Bz (>24 h), we next injected FITC-dextran (i.v.) and performed IVM to measure proinflammatory and angiogenic responses in the HCPs. Our results (Figure 5B) revealed that Bz (>24 h) restored proinflammatory indexes to baseline levels (RFU, *p* < 0.001, nVF, *p* < 0.001). In addition, the trypanocidal drug efficiently reduced two proangiogenic indexes (VA, *p* < 0.001, and H, *p* < 0.001). We next compared the complexity of the microvascular architecture of the HCPs of both groups using the PCA classifier (Figure 5B, right panel). Accordingly, 85.7% of the global indexes of infected tissues subjected to BZ treatment were positioned in the area of noninfected HCP controls (Figure 5B, right panel). Collectively, these results suggest that the complexity of the architecture of the microvascular bed of parasitized HCPs tends to be normalized by drugs that efficiently kill intracellular parasites and/or limit their transformation into intracellular trypomastigotes. LENA, we replaced Figure 5A of original MS because the formatting of columns width was lost!

### 2.5. Inflammatory Neovascularization Starts at Early Stages of T. cruzi Infection of the HCP

**Figure 6:**
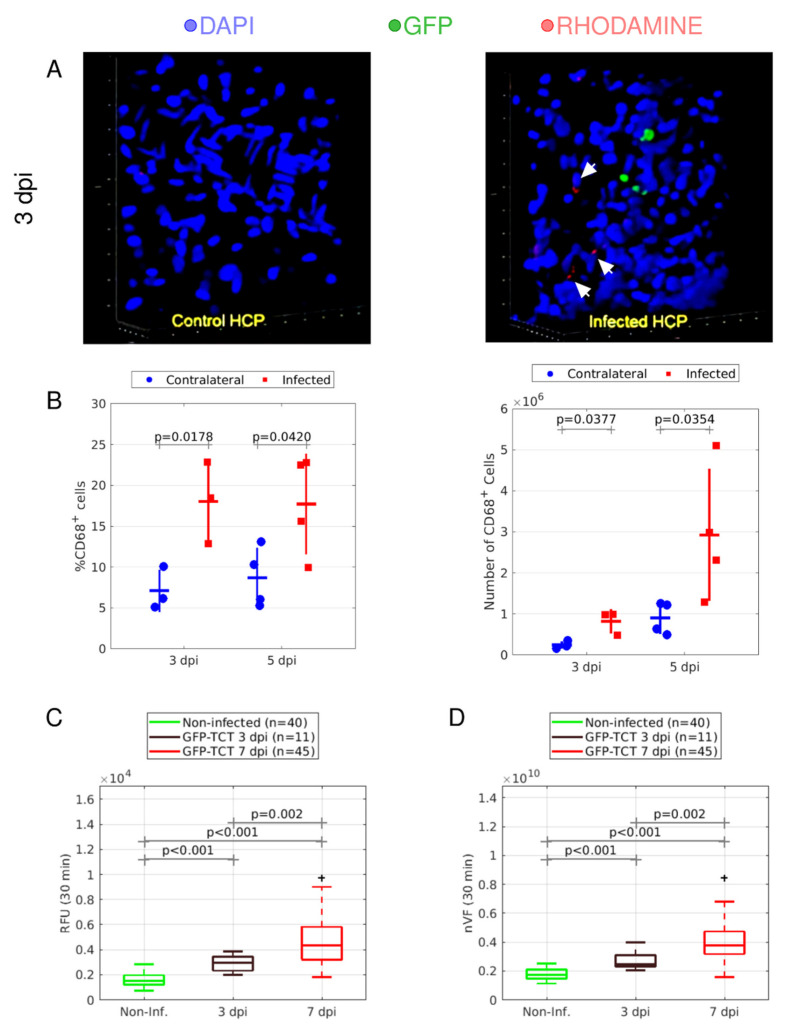
**Microvascular alterations at early stages of *T. cruzi* infection.** (**A**) Hamsters were inoculated with GFP-TCTs in the left cheek pouch. Three days later, we labelled circulating leukocytes by injecting rhodamine (red) intravenously. After visualizing leukocyte-adhesion to the endothelial lining of capillary vessels, the tissues were fixed (90 min) with formalin, stained with DAPI (blue), and analyzed by confocal microscopy. Infiltrating rhodamine (red) leukocytes were detected close to parasite nests (green) in contrast to an absence of cellular infiltrates in the parenchyma of mock-infected (PBS) contralateral HCPs. (**B**) Expression of CD68 marker of macrophages was analyzed by flow cytometry at 3 and 5 dpi. Percentage and number of CD68^+^ cells in the HCPs were analyzed at 3 dpi (*n* = 3) and 5 dpi (*n* = 4). Statistical analysis was performed by unilateral, paired *t*-test, comparing contralateral HCPs injected with PBS (**right**) and infected (**left**) HCPs. Percentage and number of CD68^+^ cells were compared at 3 dpi and 5 dpi. Bars represent standard deviation and data are representative of one experiment. (**C,D**); Box plots of two-vessel quantification indexes of inflammation (RFU and NVF) in the HCPs.

In the previous section, the studies performed in hamsters treated with Bz (>24 h) provided evidence that intracellular forms of *T. cruzi,* acting from inside out as a trigger of inflammation, might orchestrate angiogenesis. Focusing at 3 dpi, H&E staining of the HCPs (Figure S4) revealed that leukocyte infiltration was only marginally increased (*p* = 0.0730). Seeking to improve the sensitivity of this read-out, we next labeled circulating leukocytes (3 dpi) with rhodamine and maintained HCP superfusion with buffered medium during IVM. After fixing the HCP tissues at various time points, confocal microscopy images revealed that the infected tissues were infiltrated within 90 min by rhodamine-labeled leukocytes, some of which were clustered around foci of GFP-*T. cruzi* (Figure 6A, right panel). As controls, the contralateral HCPs (noninfected) were free of rhodamine-labeled leukocytes (Figure 6A, left panel). Further evidence of leukocyte infiltration was obtained following flow cytometry analysis of tissue homogenates with cross-reactive antibodies for CD68 (murine macrophage marker). As shown in Figure 6B, we found that the number and percentage of CD68^+^ cells were increased both at 3 dpi (*p* = 0.0178 and *p* = 0.0377, respectively; Figure 6B) and 5 dpi (*p* = 0.0420 and *p* = 0.0354, respectively; Figure 6B).

Having shown that circulating leukocytes transmigrate across the endothelium during the period (3 dpi) in which intracellular amastigotes replicate in permissive host cells, we next employed IVM combined to our computational methods to evaluate whether plasma (FITC-dextran tracer) leaked through postcapillary venules at this early time-point. Our results show that both RFU and nVF were increased over baseline (Figure 6C,D). As the infection proceeded, both inflammatory indexes were further boosted. Combined, these IVM observations suggest that intracellular parasites, acting from inside out, trigger plasma leakage at the expense of inflammation.

### 2.6. Vascular Remodeling Indexes Are Variable and Correlate with Transcriptional Expression of Proinflammatory Cytokines

**Figure 7:**
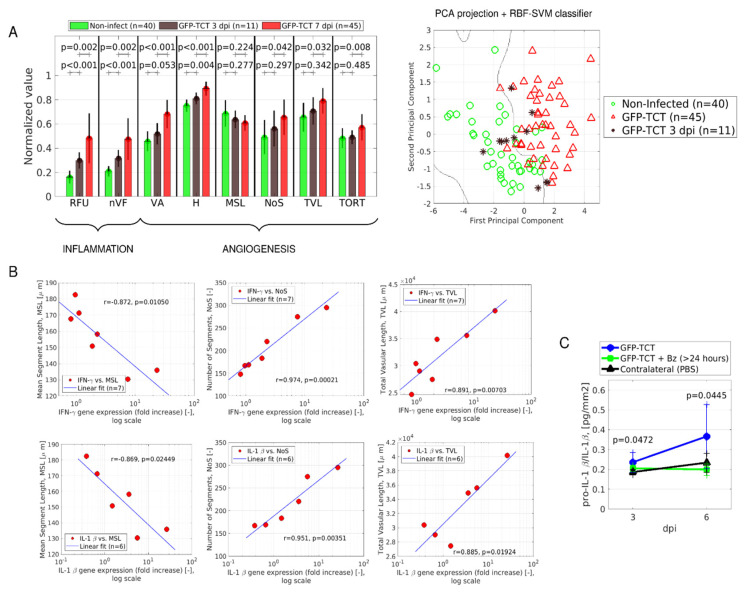
Correlation between microvascular indexes and transcriptional expression of proinflammatory cytokines at early stages of infection. (**A**) Hamsters were infected in the left HCP with 10^6^ GFP-TCTs. At the timepoint indicated, IVM was performed and the extent of inflammatory neovascularization in the HCP was characterized through eight microvascular indexes. **Left panel**: The graph illustrates responses of these indexes recorded at two different timepoints, 3 dpi (*n* = 11) versus 7 dpi (*n* = 45). The same functional parameters were measured in noninfected HCPs (*n* = 40). All hamsters were injected with FITC-dextran (i.v.) and images were recorded at 30 min after injection. The parameters quantified were RFU, nVF, VA, H, MSL, NoS, TVL and TORT. Data were analyzed with a two-tailed Mann–Whitney nonparametric test and actual *p*-values are represented for comparisons between 3 dpi (brown) versus noninfected control (green) and versus 7 dpi (red). **Right panel**: Assessment of the global complexity of the microvascular architecture at 3 dpi. The classifier algorithm PCA-RBF-SVM was used to define the decision boundary between noninfected (green diamonds) and parasitized HCPs (red diamonds). (**B**) Hamsters (*n* = 7) were inoculated (left HCP) with 10^6^ GFP-TCTs. After IVM at 3 dpi, HCP tissues were collected, and RNA was extracted to measure the transcriptional levels of IFN-γ and pro-IL-1β by qPCR. Correlation analyses were performed between transcriptional expression of IFN-γ gene (*n* = 7, fold change, top row of B) or pro-IL-1β (*n* = 6, fold change, bottom row of B) and three proangiogenic parameters: MSL, NoS, and TVL. Top row: results for IFN-γ versus MSL, IFN-γ versus NoS and IFN-γ versus TVL. Bottom row: correlation analysis of pro-IL-1β gene (fold change) versus MSL, versus NoS and versus TVL. (**C**) Two groups of hamsters (*n* = 4 each) were challenged by GFP-TCTs as described. Twenty-four hours later, we treated one group of parasitized hamsters (orally, for 2 or 5 days) with the anti-parasite drug Bz (100 mg/kg/day) and IL-1β levels in tissue extracts were determined at 3 or 6 dpi. (Pro)-IL-1β levels were measured by ELISA and levels were normalized according to the HCP area (pg/mm^2^). Baseline levels of cytokine production were measured in HCP homogenates of mock-infected HCPs (contralateral pouch inoculated 3 or 6 days earlier with PBS). Statistical analysis was performed by unilateral, paired Student’s *t*-test, comparing mock-infected (contralateral pouch inoculated with PBS) with parasitized HCPs (3 dpi) and parasitized HCPs (7 dpi) treated with Bz (>24 h) for 5 days.

In the next series of studies, we asked whether the subtle increases in microvascular permeability observed at 3 dpi translated into angiogenesis (Figure 7A). Using the same digital methods applied in the previous sections, we found that entropy was already increased (H, *p* < 0.004). Measurements of other proangiogenic indexes at 3 dpi (VA, MSL, NoS, TVL, and TORT) followed the same trend as in tissues infected 7 days earlier, although the values were not different from noninfected controls. We then used the classifier algorithm to analyze the global architecture of the microvascular beds of parasitized HCPs. Interestingly, the global indexes of individual images were positioned close to the decision boundary that separates the prototypical architecture of noninfected HCPs from those of parasitized/injured (7 dpi) tissues (Figure 7A, right panel).

Taking advantage of the phenotypic variability observed in the genetically outbred hamster cohort, we then asked whether angiogenic indexes (3 dpi) correlated with innate inflammatory responses. Indeed, using qPCR to measure IFN-γ transcription in the HPC (3 dpi), we found that mRNA levels correlated with several proangiogenic indexes: MSL (*r* = −0.872 and *p* = 0.0105), NoS (*r* = 0.974 and *p* = 0.00021), and TVL (*r* = 0.891 and *p* = 0.00703) (Figure 7B, top row). Similar to the data shown for IFN-γ, fold changes for mRNA of pro-IL-1β correlated with MSL (*r* = −0.869, *p* = 0.02449), NoS (*r* = 0.951 and *p* = 0.0351), and TVL (*r* = 0.885 and *p* = 0.01924) (Figure 7B, bottom row). As the infection progressed from 3 dpi to 6 dpi, the ELISA result showed that levels of pro-IL-1β/IL-1β in infected tissue homogenates were increased in comparison to mock-infected (PBS) contralateral tissues (Figure 7C, *p* = 0.0472). Notably, the levels of pro-IL-1β/IL-1β were decreased (*p* = 0.0445) in hamsters subjected to oral treatment for 2 days with Bz (>24 h).

### 2.7. Proteomic Analysis Identified Chymase as the Most Upregulated Proteins of the HCP at Early Stages of T. cruzi Infection

**Figure 8:**
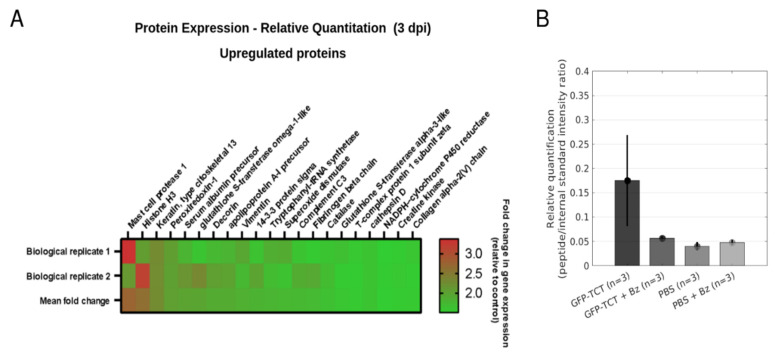
**Proteomic analyses of parasitized HCP tissues at early stages of infection.** (**A**) We compared the proteomic profiles between HCPs inoculated with 10^6^ GFP-TCTs versus HCPs of hamsters (*n* = 2) injected with PBS. At 3 dpi, the hamsters were euthanized, and the HCPs were subjected to protein extraction. Following digestion, the cleaved peptides were labeled with an isobaric tag for absolute and relative quantitation (iTRAQ). The heatmap indicates the proteins whose expression was upregulated or downregulated in the cheek pouch (< or >1.5-fold change of infection/mock-infection (PBS). Representative of one experiment. (**B**) Hamsters were infected with 10^6^ GFP-TCTs (*n* = 3) in the left HCP or injected in the contralateral pouch with PBS (*n* = 3). After twenty-four hours, the hamsters were orally treated (*n* = 3) or not (*n* = 3) with Bz (100 mg/kg/day). Graph representing targeted proteomics at 3 dpi was performed for MC protease 1 (chymase). Three prototypic peptides corresponding to MC protease 1 were analyzed in a mass spectrometer and quantified in relation to a standard peptide. Representative of one experiment with 3 animals per group. Bars represent standard deviation (SD).

Having established 3 dpi as a timepoint in which vascular remodeling was already measurable, we next compared the proteomic profile (Figure S6A,B; Table S1–S3) (Figure S6A,B; Table S1–S3) of HCPs (left, A2) infected with GFP-TCTs with the profile of PBS-injected HCPs (left, B2) excised from a normal hamster. Baseline levels of polypeptide expression in the HCPs were evaluated (S6) by comparing (i) the contralateral HCP (noninoculated, A1) excised from the infected hamster versus the profile of contralateral HCP (noninoculated, B1) of a normal hamster, (ii) PBS injected in the HCP (left, B2) of a normal hamster with the contralateral HCP (noninoculated) of the same normal animal (B1). As shown in the Venn diagram (S6A), we found 90 proteins upregulated and 17 downregulated in the parasitized HCP (A2) in comparison with PBS-inoculated HCP (B2) (Figure S6B; Table S1). Minor changes in protein expression were observed in HCPs injected with PBS in comparison with the noninoculated contralateral HCPs: 2 were upregulated and 12 were downregulated (Figure S6A; Table S2). When we compared the PBS-inoculated HCP with a contralateral HCP from another animal that was not injected with PBS, we found 10 upregulated and 23 downregulated proteins (Figure S6A; Table S3). Examples of major differentially expressed proteins in different experimental conditions are shown in the heatmap of Figure 8A.

Congruent with the increased density of MCs observed in parasitized HCPs (3 dpi; see histology data in Figure S7A,B), the MC protease-1 (MCP-1, or chymase) stood out as the most upregulated protein, with ~2.8 mean fold change in comparison with the PBS-inoculated HCPs (mock control) (Figure 8A; Table S1). Interestingly, toluidine blue staining appointed clusters of MCs degranulating at 3 dpi (Figure S7A). Notably, two plasma proteins (complement C3 and serum albumin) were also identified as major, upregulated proteins (Figure 8A; Table S1). These results support the notion that the microvasculature of the HCP is “leaky” at early stages (3 dpi) of infection (Figure 6C,D). Of note, the detection of cytokines or classical proangiogenic factors was beyond the sensitivity of our proteomic analysis.

To strengthen these molecular studies, we performed label-free, targeted proteomic analysis to quantify the peptide sequences corresponding to MCP-1(chymase) at 3 dpi. This involved comparison of the tissue levels of chymase in HCPs of naïve hamsters challenged 3 days earlier with GFP-TCTs, with a second group of infected hamsters subjected to treatment with Bz (>24 h), as well as two other control experiments. A standard peptide was equally added to the tissue samples, and chymase peptides were quantitated. The MS data (Figure 8B) represent the relative amounts of chymase based on its most intense fragments. Consistent with quantitative iTRAQ analysis, we found significantly higher levels of chymase in HCPs inoculated 3 days earlier with GFP-TCTs than in the contralateral HCPs injected with PBS (*p* = 0.0302). Notably, however, the treatment with Bz (>24 h) significantly reduced the levels of chymase in the parasitized HCPs. Of further interest, histological studies conducted after toluidine blue staining revealed that MC density was slightly increased over MC levels found in the contralateral PBS-injected pouch (Figure S7B,C).

### 2.8. Pharmacological Targeting of the MC/Chymase Pathway Blunts Inflammatory Neovascularization

**Figure 9:**
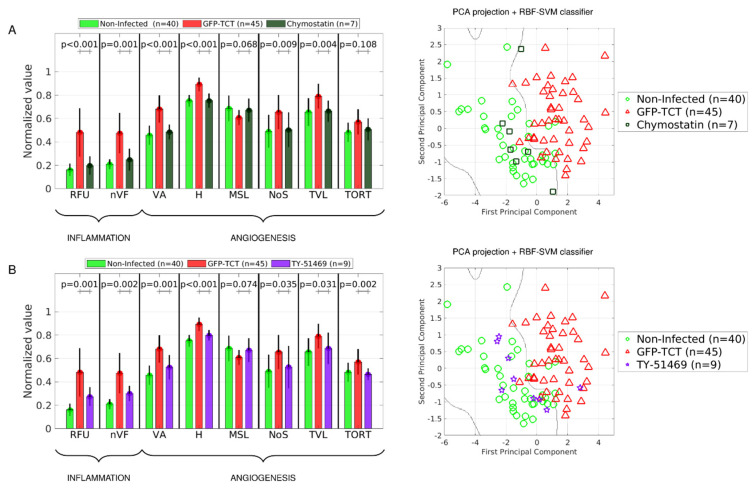
**Pharmacological targeting of mast cell chymase inhibits inflammatory neovascularization**. (**A**) **Left panel**: Microvascular indexes (RFU, nVF, VA, H, MSL, NoS, and TVL) of parasitized HCPs (7 dpi) of hamsters treated with chymostatin (*n* = 7) compared to parasitized tissues (7 dpi) (*n* = 45) and noninfected HCPs (*n* = 40). Differences between microvascular indexes are indicated by *p*-values obtained with a two-tailed Mann–Whitney test. **Right panel**: Comparison of the architectonic complexity of the microvascular beds of HCPs (7 dpi) following daily treatment with chymostatin (*n* = 7). Decision boundary between noninfected HCPs (*n* = 40; green diamonds) and parasitized HCPs (7 dpi; *n* = 45, red diamonds) was defined using the classifier algorithm PCA-RBF-SVM (**B**) **Left panel**: Microvascular indexes of parasitized HCPs (7 dpi) treated with TY-51469 (*n* = 9) compared to parasitized HCPs (*n* = 45) or to noninfected HCPs (*n* = 40). **Right panel**: Comparison of the architectonic complexity of the microvascular beds of HCPs (7 dpi) following daily treatment with TY-51469. Decision boundary defined as described for the chymostatin-treated hamster cohort.

Using a hamster sponge model of angiogenesis, Muramatsu and co-workers [56,57] demonstrated that chymase, a serine protease normally stored in MC granules, promotes angiogenesis via the angiotensin pathway. As shown above, MC density was significantly increased in the parasitized HCP (3 dpi) (Figure S7A,B). Resorting to a pharmacological approach, we next asked whether targeting of chymase could blunt inflammatory neovascularization. To this end, separate groups of infected hamsters were treated with either chymostatin or TY-51469, both extensively used as chymase inhibitors [56,58,59]. Using FITC-dextran as a tracer as in previous experiments, we found that chymostatin efficiently inhibited (Figure 9A) (i) proinflammatory indexes (RFU and nVF) (ii) four out of six proangiogenic indexes (Figure 9A, left panel). We then applied the classifier algorithm and verified that 85.7% of the global indexes yielded by chymostatin/infected HCPs were positioned in the area of noninfected controls (Figure 9A, right panel). We then extended this analysis to HCPs of animals treated systemically with TY-51469, a more selective chymase inhibitor, and found that five out of six microvascular indexes were significantly reduced (Figure 9B, left panel). Furthermore, the classifier algorithm positioned 88.9% of the global indexes of parasitized tissues of TY-51469-treated hamsters in the area of noninfected controls (Figure 9B, right panel). Taken together, the results obtained in our pharmacological studies linked the extent of inflammatory neovascularization in the parasitized tissues to the enzymatic function of chymase, a proangiogenic serine protease produced in hamster MCs [56,57].

## 3. Discussion

Decades after the disclosure of microcirculatory abnormalities in the heart autopsies of patients with chronic Chagas disease [60,61,62], it is still unclear whether the formation of dilated and tortuous microvessels in the myocardium might be a sequel of tissue parasitism and/or reflect collateral damage associated to T cell-dependent immunopathology. Unfortunately, technical obstacles preclude the use of IVM as a tool to dissect the role of microvascular alterations in heart tissues parasitized by *T. cruzi*. In the current work, we used IVM combined with proteomics and pharmacology to investigate the dynamics of inflammatory neovascularization in the parasitized HCPs. In a key observation, we verified that microvascular leakage and angiogenesis were blunted in hamsters treated with Bz, a trypanocidal drug administered 24 h after inoculation of GFP-TCTs. Initial evidence supporting a role for perivascular MCs in *T. cruzi*-induced neovascularization came from our current unbiased proteomic analysis, which identified chymase, a proangiogenic serine protease normally stored in secretory granules of MCs, as the most upregulated polypeptide in the parasitized HCPs 3 dpi. In line with this concept, our pharmacological studies revealed that infection-associated angiogenesis was reduced by two different chymase inhibitors (chymostatin and TY-51469). Since the intracellular development of *T. cruzi* is not synchronized, we may predict that angiogenesis might be further stimulated as soon as a subset of infected stromal cells die, inevitably exposing the injured tissues to proinflammatory trypomastigotes. While not excluding the possibility that the extracellular trypomastigotes may disseminate the infection via the lymphatic system, it is tempting to speculate that, like malignant tumors, these flagellated parasites may take advantage of vascular remodeling to cross the endothelium and disseminate the infection systemically.

Macroscopic observations (7 dpi) consistently showed alterations of the normal microvasculature, sometimes manifested by scattered foci of hemorrhage. As mentioned earlier, the notion that *T. cruzi* infection develops asynchronously in the HCP was supported by two lines of observation, both made at 7 dpi: First, using confocal microscopy, we found large numbers of intracellular GFP-*T. cruzi* sheltered in seemingly intact host cells. These findings imply that intracellular parasite outgrowth and/or morphogenesis are still in progress at 7 dpi, at least in a fraction of host cells. Second, using the low-resolution optical lenses that are routinely employed in IVM, we noticed that, at 7 dpi, the HCPs displayed numerous deposits of GFP. Notably, the GFP aggregates were hardly found at early stages of infection (3 dpi), and were not detected in HCPs challenged by GFP-Epis. Moreover, the GFP deposits virtually disappeared from the HCPs of hamsters that were continuously treated orally with Bz (>24 h). It is unclear whether these relatively large and densely distributed patches of GFP might have represented megacysts or consisted of tissue-infiltrating phagocytes loaded with GFP-parasites. Alternatively, the GFP aggregates observed at 7 dpi may have represented GFP fragments that had coalesced inside phagocytic vacuoles, following parasite death and proteolytic digestion of GFP protein. Although the ultrastructure of these GFP deposits remains elusive, their presence at high density at 7 dpi is an indicator of massive parasite outgrowth in the HCPs.

Although the study of innate immunity in hamsters is constrained by the lack of research tools (antibodies and transgenic animals), the finding that Bz (>24 h) dampens plasma leakage and inflammatory neovascularization strongly suggests that intracellular parasites, acting from inside out, manipulate microvascular homeostasis via activation of hitherto uncharacterized sensors of innate immunity. IVM microscopy showed that the baselines of microvascular permeability were consistently increased at early stages of (3 dpi) tissue parasitism. As predicted, at this timepoint, we found a few nests of GFP-labeled amastigotes in the HCPs, suggesting that intracellular parasites were starting binary division in subsets of permissive host cells. Importantly, two lines of evidence indicated that a low-grade inflammation was in progress at this early stage of infection: (i) rhodamine-labeled leukocytes were rapidly recruited (within 90 min) to the infection foci; and (ii) the tissues exhibited increased density of MCs. Since fibroblasts are major cellular constituents of the HCP, it is possible that alterations of microvascular homeostasis might be coordinated by neutrophil-attracting chemokines secreted by infected fibroblasts. Insight into this question comes from recent transcriptomic profiles of chemokines produced by human foreskin fibroblasts infected with either the CL Brener (virulent) or CL-14 (avirulent) *T. cruzi* clone [63]. As pointed out by the authors, the transcription of chemokine and cytokine genes was only triggered after 40 h of fibroblast infection, i.e., a timepoint that corresponds to the initial period of amastigote outgrowth. Second, they reported that CL Brenner clone (virulent) produced higher levels of neutrophil-attracting CXC chemokines (e.g., CCL2, IL-8) and G-CSF in human fibroblasts as compared with the avirulent clone. Although hamster macrophages and infiltrating leukocytes are classically responsive to intracellular parasites, it is also possible that parasitized fibroblasts (3 dpi) may adjust their metabolism by opening the endothelial barrier via upregulated secretion of neutrophil-attracting CXC chemokines. While discussing the general significance of plasma exudation in angiogenesis, Carmeliet and Jain [64] made the point that most proangiogenic factors are inducers of microvascular permeability in injured peripheral tissues. Accordingly, the influx of plasma proteins precedes thrombin-dependent formation of the provisional fibrin matrix that supports the migration of endothelial-tip cells at the onset of angiogenesis. In the absence of histological signs of tissue injury at 3 dpi, we may infer that inflammatory neovascularization in the HCP is likely initiated by intracellular parasites.

A fundamental question that comes to mind at this point is what are the benefits that intracellular amastigotes take from opening the endothelial barrier? At first sight, survival fitness of these parasites may be improved for several reasons. For example, it is possible that the trans-endothelial influx of plasma may improve delivery of transferrin/iron complexes to tissue-resident macrophages. Following the uptake of iron, activation of NADPH-oxidase upregulates production of ROS by infected macrophages, which then becomes increasingly susceptible to intracellular parasite outgrowth [65,66]. Second, the influx of plasma may fuel host/parasite metabolism during the critical period of amastigote division, hence fine-tuning the energy consumption in ways that preserve their symbiotic relationship. Circumstantial evidence supporting this hypothesis comes from RNAi data generated from *T. cruzi*-infected HeLa cells [67]. Accordingly, amastigote outgrowth depends on the function of several genes that control energy production in the mammalian host cell (e.g., fatty acid oxidation, pteridine biosynthesis, and nucleotide metabolism). Of particular interest, in vitro studies suggested that amastigote outgrowth is linked to the activation of the Akt (prosurvival) signaling pathway [67]. Among the myriad prosurvival factors that are known to prevent abortive infection by triggering the anti-apoptotic Akt pathway, bradykinin and endothelin are potential candidates [68,69] in light of evidence that intracardiac parasitism in mice is fueled via activation of bradykinin (B2) and endothelin (ET_A_/ET_B_) receptors [21,22].

As highlighted earlier in this text, selective targeting of intracellular parasites by Bz was sought by delaying the onset of oral treatment for 24 h, hence providing inoculated trypomastigotes with a comfortable time-window to adhere and invade permissive stromal cells. Notably, we maintained the selective pressure on intracellular *T. cruzi* by treating the hamsters orally for another 5 days with this trypanocidal drug. As predicted, Bz (>24 h) drastically reduced GPF clusters (7 dpi) and, concurrently, the anti-parasite drug reversed several indexes of microvascular remodeling. Collectively, these findings strongly suggest that *T. cruzi* intracellular growth/morphogenetic development is a prerequisite for the induction of inflammatory neovascularization.

Previous studies in *T. cruzi*-infected macrophages have linked production of different proinflammatory cytokines to the signaling of oligonucleotide-sensing receptors (TLR3/TLR7/TLR8/TLR9) and/or NLRP3/ASC/caspase-1 pathway [70,71,72,73,74,75]. Despite the biological variability of our outbred hamster colony, we found that mRNA levels of pro-IL-1β and IFN-γ were upregulated at 3 dpi. Interestingly, we found that the transcriptional expression of these cytokine genes was positively correlated with the indexes of non-vascular fluorescence (FITC-dextran extravasation) and endothelial remodeling (microvessel sprouting). Notably, however, IL-1β levels were not significantly elevated at 3 dpi, despite the fact that transcription of pro-IL1-β and INF-γ genes was upregulated at this early timepoint. Interestingly in this context, Oliveira et al. [63] did not find in vitro evidence of fibroblast cell death up to 4 dpi. Notably, NLRP3 was not expressed, and congruently with this, IL-1β was not detected in the cultures of infected fibroblasts [63]. Since hamster fibroblasts are major constituents of the HCP stroma, low-level expression of NLRP3 may favor intracellular parasite outgrowth early in infection. Alternatively, NLRP3 might be expressed in the infected fibroblasts, but the levels of ATP and other damage-associated molecular patterns (DAMPs) (second signals) generated in the tissue stroma may not reach the activation threshold of inflammasomes [75].

**Figure 10:**
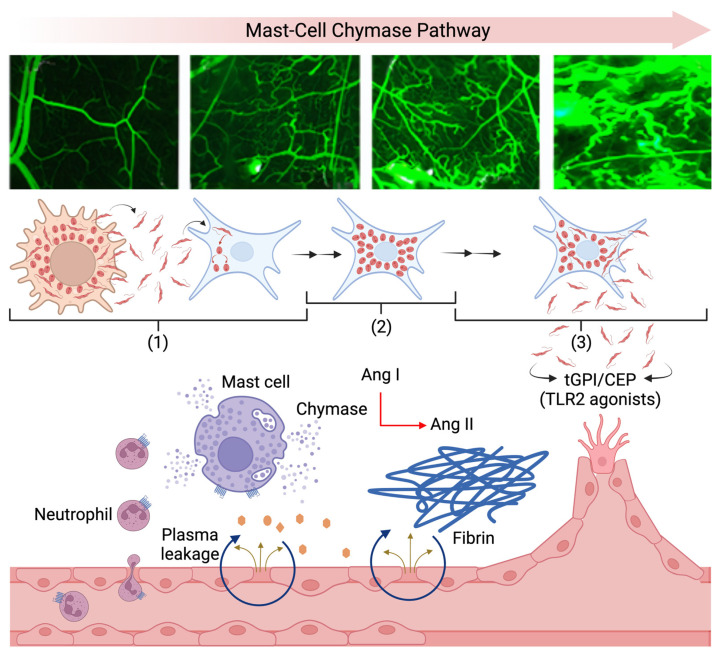
**Dynamics of infection-associated inflammatory neovascularization in the HCP.** The scheme illustrates the reciprocal benefits that infection-associated inflammatory neovascularization may bring to the host-parasite relationship during a single cycle of *T. cruzi* development in the HCP. (**1**) Following the egress of trypomastigotes from a dying, infected hamster cell (e.g., infiltrating activated macrophage), the infective parasites invade a subset of as-yet unidentified stromal cell, and start to proliferate by binary division as intracellular amastigotes. (**2**) Sensed by hitherto uncharacterized mechanisms, the replicating pathogens elicit a low-grade inflammation, manifested by microvascular leakage, leukocyte infiltration and increased density of perivascular mast cells. Following mast degranulation, plasma extravasation enables the formation of the provisional fibrin matrix that sustains the migration of endothelial tip cells at the onset of angiogenesis [64]. Analysis of the role of cardiac mast cells in animal models of heart inflammation linked fibrosis to the secretion of chymase, a proangiogenic serine protease that generates angiotensin (Ang) II independently of ACE, in humans, dogs and hamsters, [44,45,47,49]. Based on transcriptomic data obtained in cultures of *T. cruzi*-infected fibroblasts and HeLa cells [63,67], we hypothesize that intracellular amastigotes, acting from inside out, manipulate inflammation to enhance the trans-endothelial delivery of blood-borne nutrients and prosurvival factors to the foci of infection. (**3**) Once faced with the dense network of capillary vessels formed by angiogenesis, the extracellular trypomastigotes may disseminate the infection systemically after infecting microvascular cells [11] or crossing the endothelial lining [79,80,81]. Created with BioRender.com, accessed on 3 October 2016.

Using animal models of tumor-induced angiogenesis, West and co-workers [76] reported a decade ago that carboxy-ethyl-pyrrole (CEP), an oxidized lipid of endogenous origin (DAMPs), promotes TLR2-dependent angiogenesis. More recently, McCoy et al. [77] used a transgenic mouse with conditional knockout of TLR2 in endothelial cells to demonstrate that CEP (and/or other endogenous ligands of TLR2) promotes angiogenesis by stimulating the recruitment of pro-tumorigenic leukocytes to the tumor microenvironment. Given that extracellular TCTs shed extracellular vesicles bearing tGPI-mucins (TLR2 agonists) [18,19,78], it is possible that TR2-expressing endothelial cells are jointly activated by tGPI (virulent factor) and endogenous CEPs generated by infiltrating neutrophils [76,77]. Although angiogenesis in *T. cruzi*-infected tissues differs in several aspects from the settings of tumor biology, it will be interesting to know whether the released trypomastigotes might benefit from plasma leakage [22] and TLR2-dependent neovascularization [77] to spread the infection systemically [79,80,81] (Figure 10).

Strategically positioned in perivascular regions, MCs are members of a highly heterogeneous group of innate sentinel cells that contribute to tissue remodeling by releasing a broad array of proangiogenic mediators, such as VEGF, TGF-β, TNF-α, and chymase [43]. We initially turned our attention to MCs, because there was an early (3 dpi) increase in the density of these innate sentinel cells, which predominantly localized to perivascular regions (Figure S7). Exploratory proteomics (3 dpi) did not detect VEGF or other proangiogenic factors in the tissue homogenates. Strikingly, the hamster chymase, a proangiogenic serine protease normally stored in MC granules [56,57], emerged as the most upregulated polypeptide in infected HCPs. Next, we employed targeted quantitative proteomics and found that the expression levels of chymase were normalized upon oral treatment (2 days) with Bz (>24 h). Combined, these findings suggest that alert signals produced in parasitized HCPs may either stimulate the proliferation of MC precursors and/or upregulate chymase expression levels in fully differentiated MCs. Interestingly from the perspective of the pathogenesis of Chagas disease, MC density is increased in biopsies from the gastrointestinal mucosa of chronically infected patients afflicted with digestive megasyndromes [82]. Beyond the role that the MC-chymase pathway may play in the pathogenesis of digestive megasyndromes, studies in animal models of CCC may clarify whether chymase secreted by cardiac MCs may worsen myocardial fibrosis. This hypothesis is attractive because studies in the sponge model of angiogenesis revealed that hamster chymase converts angiotensin-I into the proangiogenic/profibrotic angiotensin II independently of ACE [83,84]. Intriguingly, comparative analysis of the substrate specificity of MC serine proteases from different animal species showed that chymase from humans, hamsters, and dogs is able to convert angiotensin I into the profibrotic angiotensin II, whereas some of the orthologous MCPs from mice conversely degrade angiotensin II [47,83].

Although the time window of the current infection model was limited to a single cycle of intracellular parasite development in the HCP, we showed that inflammatory neovascularization was blunted upon treatment with two chymase inhibitors, chymostatin [56] or TY-51469 (higher specificity) [85], both extensively used by other groups [84]. Given the precedent that chymase inhibitors mitigate fibrosis in animal models of inflammatory heart diseases [49,84], preclinical studies may clarify whether selective chymase inhibitors, such as TY-51469, may have therapeutic value in chronically infected hamsters and dogs, which faithfully reproduce the clinical features of the human CCC [50,51].

In summary, our findings suggest that *T. cruzi,* acting from inside out, coopts mast cells to promote microvascular leakage and steer angiogenesis via the chymase-dependent pathway. Future studies may clarify whether drug targeting of proangiogenic chymase might blunt angiotensin II-dependent heart fibrosis in the hamster model of CCC.

## 4. Materials and Methods

### 4.1. Parasites

Mammalian cell tissue culture-derived trypomastigotes (TCTs), clone Dm28c (DTU-I) [86], were harvested from the supernatants of rhesus-kidney epithelial LLC-MK2 cells (ATCC, Manassas, VA, USA), 5 dpi. Genetically modified *T. cruzi* (luc-TCT and GFP-TCTs) were developed using the integrative pTREX vector, as described [87,88]. Infected LLC-MK2 cultures were maintained in Dulbecco’s Modified Eagle Medium (DMEM), with 2% heat-inactivated fetal calf serum (FCS). At 6–7 dpi, the freshly released trypomastigotes were harvested from the culture supernatants and washed twice by centrifugation with excess Hank’s balanced saline solution (HBSS), pH 7.2. After washing, TCTs were suspended in PBS. Cultures of GFP-TCTs and luc-TCTs (Dm28c) were handled similarly to WT-TCTs (Dm28c).

### 4.2. Animals and Infection Procedures

Hamsters (Anilab, São Paulo, Brazil) were maintained and anesthetized according to regulations approved by the local ethical committee of the Federal University of Rio de Janeiro (CEUA-CCS, license #158/18), following guidelines of the Conselho Nacional de Controle da Experimentação Animal (CONCEA). Altogether, we used 114 hamsters with a mean bodyweight of 120 ± 10 g. In this cohort, 40 hamsters were used as noninfected control. Anesthesia was performed by intraperitoneal injection of ketamine and xylazine, and supplemented with i.v. α-chloralose (2.5% *w*/*v*, solution in saline) through a femoral vein catheter. A tracheal cannula (PE 190) was inserted to facilitate spontaneous breathing, and body temperature was maintained at 37 °C by a heating pad monitored with a rectal thermistor. A parasite suspension in PBS (100 µL containing 10^6^ of GFP-TCTs or GFP-Epis) was inoculated in the left cheek pouch of anesthetized hamsters using an insulin-dosing syringe, as previously described [54]. As controls, uninfected HCPs were prepared for IVM exactly as for parasitized tissues. In key experiments, as indicated in the main text, we included an internal control group in which the contralateral pouch (right) was injected with 100 µL PBS. In order to inoculate the suspension of parasites, the cheek pouch was everted and mounted as described in the routine preparation for IVM (without dissection of the tissues). The superior layer of the cheek pouch was elevated with a pair of tweezers. Using a stereomicroscope, we gently deposited the PBS suspension of the parasites (or PBS in the contralateral HCPs) in the non-vascularized space that separates the superior and the inferior part of the everted tissue, without causing visible bleeding. Following this step, the HCP tissue was then reintroduced to its normal position in the oral cavity of the anesthetized hamster.

### 4.3. Intravital Microscopy

The hamster cheek pouch was prepared for intravital microscopy as reported by our group [8,20,21,54,89], using a digital camera (AxioCamHRc, Carl Zeiss, Oberkochen, Germany). The microcirculation was observed with an Axioskop 40 microscope, using objectives of 4×, 20× (water immersion lens) and oculars 10× (Carl Zeiss, Oberkochen, Germany). The microscope was equipped with appropriate filters (490/520 nm, FITC-dextran, and 540/580 nm, TRITC-dextran), a mercury arc lamp, and a Colibri 2 LED-light source (Carl Zeiss, Oberkochen, Germany). The image resolution is 1388 × 1040 for an area of approximately 5 mm^2^ (objective 4×), which yields pixel spacing of 1.862 µm, and therefore a pixel area of 3.467 µm^2^. We used two macromolecular markers of equal molecular masses, FITC-dextran, and TRITC-dextran (150 kDa, 100 mg/kg b.w., TdB Labs, Uppsala, Sweden). TRITC-dextran was used to identify intracellular GFP-*T. cruzi* and localize microvessels by combining IVM to confocal microscopy. A digital camera, AxioCamHRc, and a computer with the AxioVision 4.4 Software program (Carl Zeiss, Oberkochen, Germany) were used for image recording and measurement of fluorescence (Relative Fluorescent Units, RFU).

### 4.4. Detection of Leukocyte Infiltration

Hamsters were infected with GFP-TCTs as previously described. Seventy-two hours later, the animals were prepared for IVM before receiving an intravenous injection of rhodamine (10 µg/kg of body weight, TdB Labs, Uppsala, Sweden) through a catheter introduced in the femoral vein [8,90]. Leukocyte rolling was observed by IVM and rhodamine was intravenously injected every 10 min [90]. After 90 min of observation, the HCP tissues were excised and fixed in paraformaldehyde solution (4%) for 24 h before being stained by 4′,6-diamidino-2-phenylindole (DAPI, 1 µg/mL, Sigma-Aldrich) for 5 min. After three cycles of washing of 3 min each, the HCP tissues were adhered to glass slides, and n-propyl-gallate (Sigma-Aldrich) was added as an anti-fading medium. Images were acquired on a Leica confocal microscope.

### 4.5. Microvascular Image Analysis

Methods for automatic characterization of the HCP microvasculature using IVM were originally described by Bulant et al. [54]. The automatic segmentation of microvascular networks resulted in a binary image, which allowed us to compute the network of vessels present in the image. Nodes (bifurcations) and edges (vessels) were identified and composed the skeleton of the network of vessels. We then measured the length and diameter of vessels, as described above [54]. The image-processing pipeline employed here involved the computation of eight microvascular indexes, namely: (1) relative fluorescence units (RFU), total fluorescence in the image; (2) Shannon’s entropy (H), statistical measure of randomness of the image, computed from the image histogram; (3) non-vascular fluorescence (nVF), total fluorescence of the image background; (4) vascular area (VA), total area of pixels corresponding to micro vessels, <100 µm in diameter; (5) number of segments (NoS), total number of arteriolar, capillary, and venular segments; (6) total vessel length (TVL), sum of all arteriolar, capillary, and venular segment lengths in the image; (7) mean segment length (MSL), average length of all arteriolar, capillary, and venular segments in the image; and (8) tortuosity (TORT), average tortuosity of all arteriolar, capillary, and venular segments in the image. It is worth pointing out that RFU and H are computed directly over the image without any further processing. Conversely, nVF and VA depend on the image segmentation. Finally, NoS, TVL, MSL, and TORT indexes depend on skeletonization of the segmented image. The pipeline for the quantification of geometric microvascular indexes is illustrated in Figure S8. To characterize angiogenic effects on captured images, we employed microvascular indexes VA, H, NoS, MSL, TVL, and TORT to build a supervised classifier using cohort samples formed by noninfected HCPs (*n* = 40) and parasitized HCPs (7 dpi) (*n* = 45). First, we applied a logarithmic transformation to the indexes and normalized the outcome by removing the mean and dividing by the standard deviation of the sample. A linear Principal Component Analysis (PCA) algorithm was then applied to reduce the dimensionality of the problem. In the subsequent analysis we considered the first two PCA dimensions (first and second principal components, which account for 72.7% and 15.8% of total variance, respectively). These first two features encode, through linear combinations of the six microvascular indexes, the information contained in the vascular architecture of the HCP. The two-dimensional (2D) space formed by the first and second principal components was used to construct a classifier based on a Support Vector Machine with Radial Basis Function (RBF) kernel. The classifier defined a decision boundary which separated the 2D PCA space into noninfected-like microvascular networks and microvascular networks of parasitized HCPs (7 dpi), referred to for simplicity as noninfected and GFP-TCT subdomains. The classifier was then applied to analyze in a blind manner the different sets of HCP images captured by IVM. A detailed description of PCA and RBF-SVM methods is described by Hastie et al. [91].

### 4.6. Pharmacological Interventions

Four drugs with completely different pharmacological characteristics were used to investigate infection-associated angiogenesis in the HCP. Benznidazol (Bz), a drug currently used in the treatment of acute Chagas disease [92], was administered orally (100 mg/kg/day), from 1 to 5 dpi starting 24 h after parasite inoculation in the HCP. Targeting of chymase, a serine protease that promotes angiotensin-1-dependent angiogenesis [56,57,93], involved daily injection (i.p.) of two drugs: chymostatin (1 mg/kg, q.d., for 4 days) and TY-51469 (20 mg/kg, q.d., for 4 days) [85], kindly donated by Prof. Shinji Takai, Department of Pharmacology and Biomedical Computation, Osaka Medical College, Takatsuki, Japan).

### 4.7. Quantitative Proteomic Analysis (iTRAQ)

Syrian hamsters (*Mesocricetus auratus*) were injected with 10^6^ GFP-TCTs. The animals were divided into two experimental groups (two hamsters per group): The experimental design involved (i) GFP-TCTs injected in the left HCPs, whereas the contralateral HCPs of the same hamsters remained intact, (ii) PBS injected in the left HCPs, whereas the contralateral HCPs of the same hamsters remained intact. Next, we excised the left and right HCPs of both groups at 72 h post-infection (hpi). The HCPs were gently washed with PBS, immediately stored in plastic tubes and frozen in liquid nitrogen. For protein extraction, the samples were thawed, weighted (100–120 mg), and homogenized using an electric tissue homogenizer. The homogenization was performed in a buffer containing urea (7 M), thiourea (2 M), sodium deoxycholate (2%), and 0.1 M triethylammonium bicarbonate buffer (TBB, Sigma-Aldrich, St. Louis, MO, USA). The tissue–buffer proportion used corresponded to 5 µL per 100 µg tissue. After homogenization in the described buffer, the samples incubated for 1 h (4 °C), followed by centrifugation (20,800× *g* for 30 min, 4 °C). The protein-rich supernatant was removed, and proteins were precipitated by the addition of four volumes (80%) of ice-cold acetone and incubation overnight at 4 °C. The protein pellet was washed twice using ice-cold acetone and air-dried in ice for the elimination of acetone. Proteins were solubilized in a buffer containing 7 M urea, 2 M thiourea, and TBB. Protein amounts were determined according to the instructions of Qubit kit (Invitrogen) and protein integrity was confirmed by gel electrophoresis. Disulfide bonds were reduced (10 mM1,4-dithiothreitol, 30 min, 30 °C), alkylated (40 mM iodoacetamide, 30 min, room temperature (rt), protected from light), and proteins digested with trypsin (Sequencing-Grade Trypsin, Promega, Madison, WI, USA) (1 µg trypsin per 50 µg protein, 18 h, 35 °C). Before trypsin digestion, the volume was adjusted with deionized water in order to achieve a tenfold higher final volume, with pH ~8. Trypsin digestion was interrupted by the addition of trifluoroacetic acid in a final concentration of 0.1% (pH~2). Following trypsin digestion, peptides were purified in a C18 MacroSpin Column (Harvard Apparatus, Holliston, MA, USA) and redissolved in deionized water, and the number of purified peptides was determined. Ensuing peptides (~33 µg per condition) were labeled with the isobaric tag for relative and absolute quantitation (iTRAQ 4 plex, ABSciex, Taipei, Taiwan), accordingly. Briefly, TBB was added to the samples in order to achieve the optimal pH 8, and labeling happened in the proportions of 30% of the sample (aqueous phase) to 70% of the organic phase (ethanol). The iTRAQ reagents were incubated for one hour with the samples at rt. iTRAQ markers 114, 115, 116, and 117 were distributed as two biological replicates according to the following experimental design: (i) infected HCP (left, 3 dpi), (ii) contralateral (noninoculated) HCP of the hamster that was challenged in the opposite (left) pouch with PBS. The reaction was stopped by acidification and the achievement of equal proportions of the organic and aqueous phase, through the addition of 0.1% formic acid. The final pH was approximately 2. After labeling, the peptides were vacuum-dried and redissolved in 10 mM KH_2_PO_4_ and 25% acetonitrile (ACN), pH 3, for cation-exchange and unbound iTRAQ removal. The cationic exchange was conducted in a proper MacroSpinColumn (Harvard Apparatus, Holliston, MA, USA), and the samples were separated in different fractions according to the concentrations of KCl used for elution (500 mM, 250 mM, 100 mM, and Flow-Through). After the cationic exchange, iTRAQ labeled peptides were purified in a C-18 Spin Column and resuspended in 0.1% formic acid. After peptide quantification, the concentration was adjusted for 0.5 µg/µL and the analysis was performed in an LTQ Velos Orbitrap (Thermo Scientific, Waltham, MA, USA) mass spectrometer. Each sample (biological replicate) was analyzed by three technical replicates. Proteins were identified using the translated genome of *Mesocricetus auratus* available on NCBI (ftp://ftp.ncbi.nlm.nih.gov/genomes/Mesocricetus_auratus/protein/), downloaded on 3 October 2016. The analysis was performed using the Isobaric Analyzer available on the software Pattern Lab for Proteomics by selecting the two conditions experiment mode. The following statistical parameters were selected: *p* = 0.05, only unique peptides, at least one unique peptide, and fold-change cutoff was set to 0.4. Proteins considered downregulated or upregulated in each comparison and in both biological replicates were used for the final result. This experiment has been registered on PRIDE repository as “Proteomic analysis of *T. cruzi* experimentally infected hamster cheek pouch”, with project accession PXD026905.

### 4.8. Target Proteomics for Chymase Quantitation

Syrian hamsters were divided into four experimental groups (three hamsters per group): (i) injected with GFP-TCTs in the left HCP; (ii) injected with GFP-TCTs in the left HCP and treated with Bz (100 mg/kg/day, oral route, starting 24 h post-infection); (iii) mock-infected (PBS-injected in the left HCP); and (iv) mock-infected and Bz-treated. The HCP tissues were excised at 72 h post-infection. Proteins were extracted from the HCPs, quantified, and trypsin-digested as previously described for the iTRAQ proteomics approach. Label-free LC-MS/MS was performed in triple-quadrupole TSQ Quantiva (Thermo Fisher Scientific) coupled to a nanoLC easy II HPLC. Samples (0.5 µg protein/µL) were subjected to chromatography performed using a gradient of A (5% ACN and 0.1% formic acid) and B (95% ACN and 0.1% formic acid). Samples were run at a flux of 320 nL/min. Quadrupoles I and III of the triple-quadrupole were set to 0.7. Quantitation was performed by the addition of a standard peptide: EVELLNEK (972.5200 Da). The standard peptide was modified by the addition of carbon-13 and nitrogen-13 in the lysine residues. The following mast cell protease 1 peptide sequences were analyzed in the MS: GDAKPPAVFTR (1158.6265 Da), GDSGGPLLCAGVAHGIVSYGR (1985.861 Da), and GFTASCGGFLITPEFVMTAAHCK (2388.1185). The peptide sequences described can be found in sequence NP_001297495.1 (Mast cell protease 1 precursor—*Mesocricetus auratus*).

### 4.9. Histopathological Analysis

Hamster cheek pouches were excised and fixed in buffered 5% formalin solution (pH 7.4) as described [94]. After fixation, tissues were paraffin embedded. Pouch sections of 5 µm were obtained in a paraffin microtome, clarified, and hydrated for staining with Hematoxylin & Eosin and Toluidine Blue. Inflammatory infiltrates were analyzed from ten fields using the software Image J for quantification of the area occupied by cell nuclei [95]. Mast-cell density was determined by manual counting of this cell type in all fields of HCP tissue sections. Mean values obtained from total counting were used for statistical analysis. Contralateral pouches and HCP tissues inoculated with GFP-TCTs were compared by paired *t*-test.

### 4.10. Flow Cytometry Analysis

Hamsters were infected as previously described and, after three or five days, the animals were euthanized and the cheek pouch tissues were digested by incubation (1 h, 37 °C) in 5 mL serum-free DMEM containing 500 µg/mL type IV collagenase (Sigma-Aldrich, St. Louis, MO, USA) and 500 µg/mL hyaluronidase (Sigma-Aldrich, St. Louis, MO, USA). The digestion was stopped by the addition of DMEM containing 10% FCS (30 μL, per sample). Tissue debris was removed using cell strainers (40-µm pore). Cell suspensions were centrifuged (350× *g*, 15 min), and the cellular suspension was stained with PE anti-mouse CD68 antibody (Biolegend clone FA-11) diluted at 1:100 dilution or incubated with same dilution of matched PE Rat IgG2a κ isotype (Pharmingen, San Diego, CA, USA). Flow cytometry was performed in a FACSCalibur flow cytometer (BD Biosciences, San Jose, CA, USA). Summit software was used for dot-plot analyses. The percentage and absolute numbers of CD68^+^ cells found in the contralateral and parasitized HCPs were compared and statistical analysis was performed using Student’s *t*-test.

### 4.11. Bioluminescence Experiments

Hamsters were injected with an inoculum containing 10^6^ TCTs expressing GFP protein and 10^6^ luc-TCTs in the HCP. After 7, 14, or 21 dpi, the animals were intravenously injected with D-luciferin (Caliper), 150 mg/kg body weight, as previously described [87]. Images were obtained in a bioluminescent imaging system (IVIS-Lumina; Caliper, Hopkinton, MA, USA) and bioluminescence was measured 5, 10, and 15 min after D-luciferin injection. Maximum bioluminescence levels were obtained 10 min after D-luciferin intravenous injection, and this time was used for analysis. Following analysis in the HCP, different organs and tissues were isolated for visualization of parasite bioluminescence: heart, salivary lymph nodes, testis, masseter muscle, and HCP fat. The minimum of 600 counts was used as a technical threshold for bioluminescence detection, and results are expressed in radiance as shown in picture scales.

### 4.12. Quantitation of Parasite Load in the Tissues

Parasite load was analyzed by quantitative real-time polymerase chain (qPCR) reaction. The DNA extraction was performed using DNAeasy (QIAGEN) extraction kit for obtention of DNA from tissue and blood samples, as previously described [96]; 20 or 100 ng of tissue DNA were used for PCR reaction. qPCR was performed using the SYBR Green probe (Life Technologies, Carlsbad, CA, USA). Amplification was performed according to the following program: 95 °C (10 min), 95 °C (30 s—45 cycles) and 60 °C (1 min). The described PCR steps were followed by a denaturation curve. The following primer sequences were used for qPCR reaction in the concentration of 0.4 µM: GCTCTTGCCCACACGGGTGC (forward) and CCAAGCAGCGGATAGTTCAGG (reverse). A standard curve, ranging from 2.5 to 2.5 × 10^5^ of GFP-TCT parasite equivalent of DNA, was used to convert Ct values into the number of parasites/mg of tissue.

### 4.13. Cytokine Gene Expression

Gene-expression fold change of parasitized HCP (left) was quantified using mock-infected tissues as controls (contralateral pouch inoculated with PBS). RNA extraction was performed at 3 dpi according to the protocol described [97]. RNA (2 µg) was used for cDNA synthesis reaction according to the Reverse Transcription kit provided by Life Technologies. The cDNA amount was determined by a fluorometric assay using Qubit single-strand DNA assay kit and 20 ng of cDNA were used in qPCR reactions. The primers for quantitation of IFN-γ were previously described [97]. The primers used for the detection of pro-IL-1β were GGCTGATGCTCCCATTGC (Forward) and CACGAGGCATTTCTGTTGTTCA (Reverse). Data were analyzed using Thermo Fisher Scientific Dashboard available online.

### 4.14. ELISA for IL- 1β Quantitation

After 3 or 6 dpi, HCP tissues were excised and protein extraction was performed using a tissue homogenizer in a buffer containing PBS, 0.1% Triton X-100, and a protease inhibitor cocktail (PIC) (Roche) (PTP buffer). Tissues were homogenized in 500 µL PIC buffer. ELISA assay was performed according to information provided by the R & D mouse IL-1β ELISA development system. IL-1β-detected amounts were normalized according to the tissue area of the HCP (318,746 mm^2^), as protein content and weight change in infected tissues.

### 4.15. Statistical Analysis

Intravital microscopy data were analyzed by the nonparametric Wilcoxon–Mann–Whitney test. Comparisons between contralateral and parasitized HCP tissues were performed using unilateral or bilateral paired *t*-test, as described in legends of figures. We have adopted the criteria proposed by Amrhein et al. [98], showing actual *p*-values. Graphs and statistical tests were performed using MATLAB version 9.5.0 (R2018b, Natick, MA, USA: The MathWorks Inc.).

## Data Availability

Data referring to quantitative proteomic analysis were registered on PRIDE repository as “Proteomic analysis of T. cruzi experimentally infected hamster cheek pouch”, with project ac-cession PXD026905.

## Supplementary Materials

The following supporting information can be downloaded at: https://www.mdpi.com/article/10.3390/pathogens11020187/s1, Figure S1: Comparison of microvascular indexes in left (infected) and contralateral (noninfected) HCP, Figure S2: Dose-response dependency of microvascular responses elicited by GFP-T. cruzi, Figure S3: Comparison of microvascular indexes between PBS-injected HCP and noninfected controls, Figure S4: Histopathological analysis of HCP at early stages of infection, Figure S5: Cytokine expression levels in the parasitized HCP, Figure S6: Venn diagram of proteomic analysis, Figure S7: Mast cell density in parasitized HCP, Figure S8: Image processing methodology. Table S1: Protein relative quantitation by LC-MS/MS of the experimental comparison between GFP-TCT-inoculated HCP (left) vs. contralateral (noninoculated) of normal hamster, Table S2: Protein relative quantitation by LC-MS/MS of the experimental comparison between PBS-inoculated HCP of normal hamster vs. contralateral (noninoculated) of normal hamster, Table S3: Protein relative quantitation by LC-MS/MS of the experimental comparison between contralateral HCP excised from an infected hamster vs. contralateral (noninoculated) HCP of normal hamster.
