# Supplementary material for: Sheltered in Stromal Tissue Cells, Trypanosoma cruzi Orchestrates Inflammatory Neovascularization via Activation of the Mast Cell Chymase Pathway
Source: Pathogens. 2022 Jan 29;11(2):187. doi: 10.3390/pathogens11020187 (PMC8878313; doi:10.3390/pathogens11020187)
Supplement: Supplementary file 1 [file pathogens-11-00187-s001.zip › Supplementary -08 Feb2022-reviewed S7.pdf]

Supplementary Information

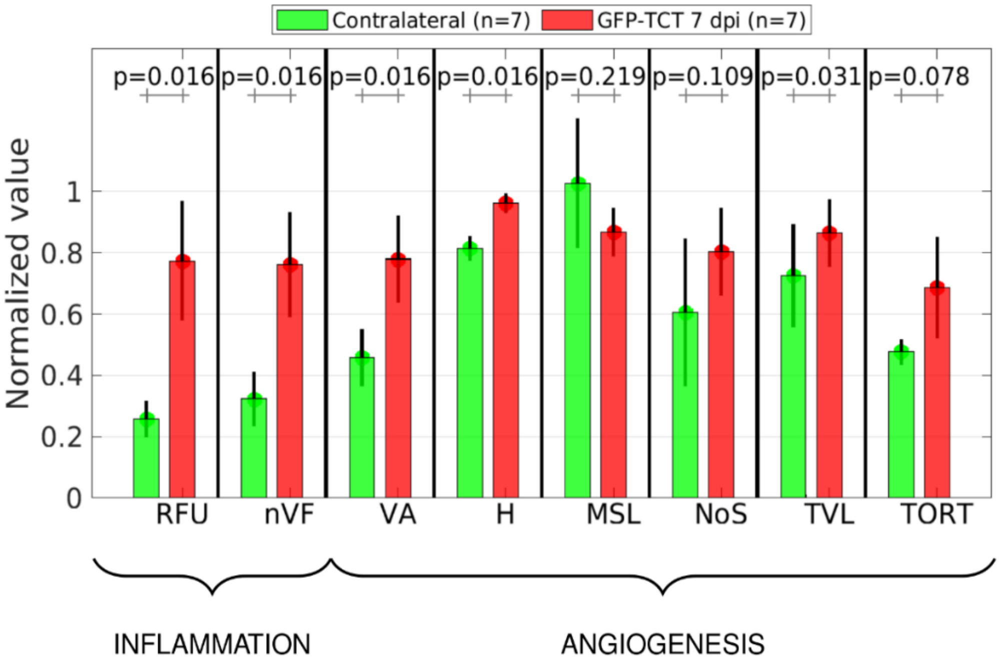

S1 Fig. Comparison of microvascular indexes in left (infected) and contralateral (noninfected) HCP. IVM measurements of the proinflammatory (RFU, nVF) and proangiogenic (VA, H, MSL, NoS, TVL, TORT). Indexes of parasitized HCP (7dpi) are compared with the profile of noninfected contralateral pouch of the same animal (n = 7). p-values of pairwise two-tailed Mann-Whitney test for differences between left and contralateral HCP (n = 7) are inserted.

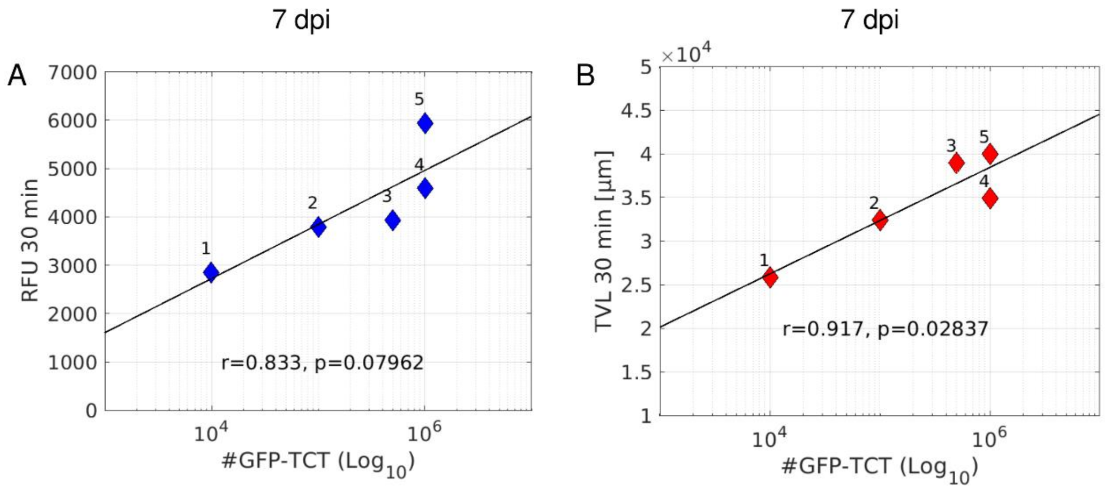

S2 Fig. Dose-response dependency of microvascular responses elicited by GFP-*T. cruzi*. The hamsters (n = 5) were injected in the left cheek pouch with different inoculums 10^4 (1), 10^5 (2), $5x10^5$ (3), and 10^6 (4, 5) GFP-TCTs. HCPs were subjected to IVM at 7 dpi and the following microvascular parameters were analyzed: RFU **(A)** and TVL **(B).** Pearson's correlation coefficients (*r*) and p-values were calculated for correlations between RFU and inocula of TCT-GFPs (log scale), and TVL and inocula of TCT-GFPs (log scale).

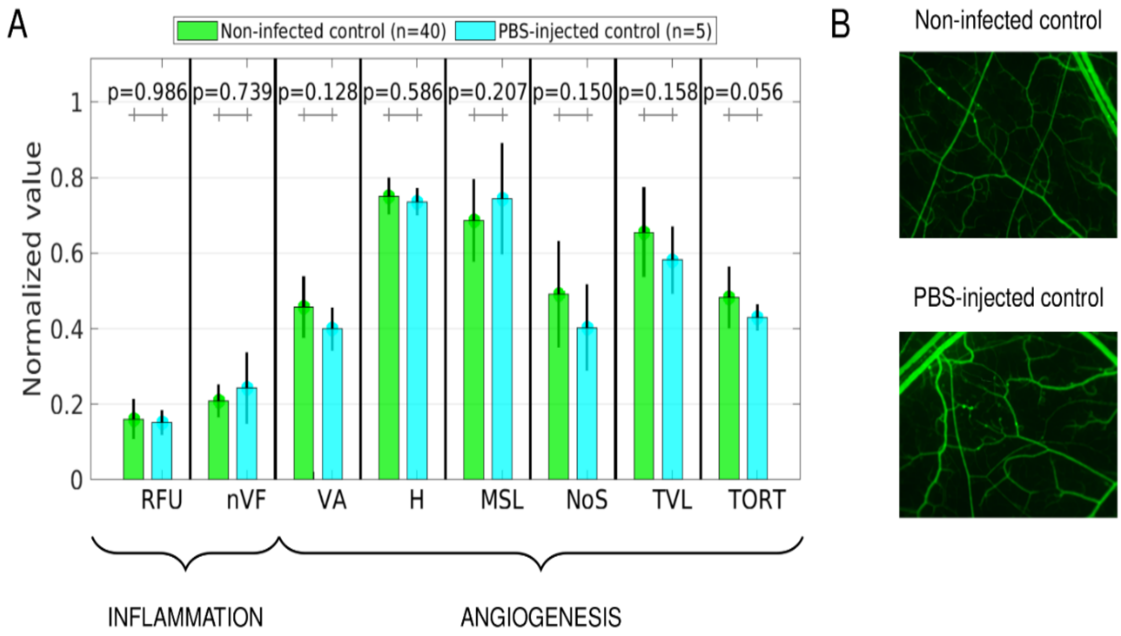

S3 Fig. Comparison of microvascular indexes between PBS-injected HCP and noninfected controls. (**A**) Quantification of eight microvascular indexes between contralateral HCP (right) injected 7 days earlier with PBS (n = 5) and noninfected HCP (left) controls (n = 40). **(B)** Image captured by IVM illustrates a typical profile of FITC-dextran tracing of noninfected HCP, and of a representative image of the contralateral (bottom) inoculated with PBS.

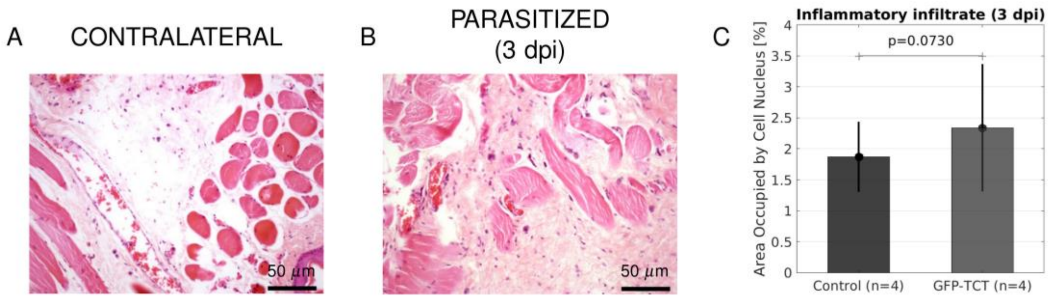

S4 Fig. Histopathological analysis of HCP at early stages of infection. (A-B) Inflammatory infiltrates were quantified at 3 dpi. **(C)** Scores were obtained analyzing the area (mean ± SD) occupied by cell nucleus (contralateral and infected) and statistical analysis was performed by paired *t*-test (p = 0.0730).

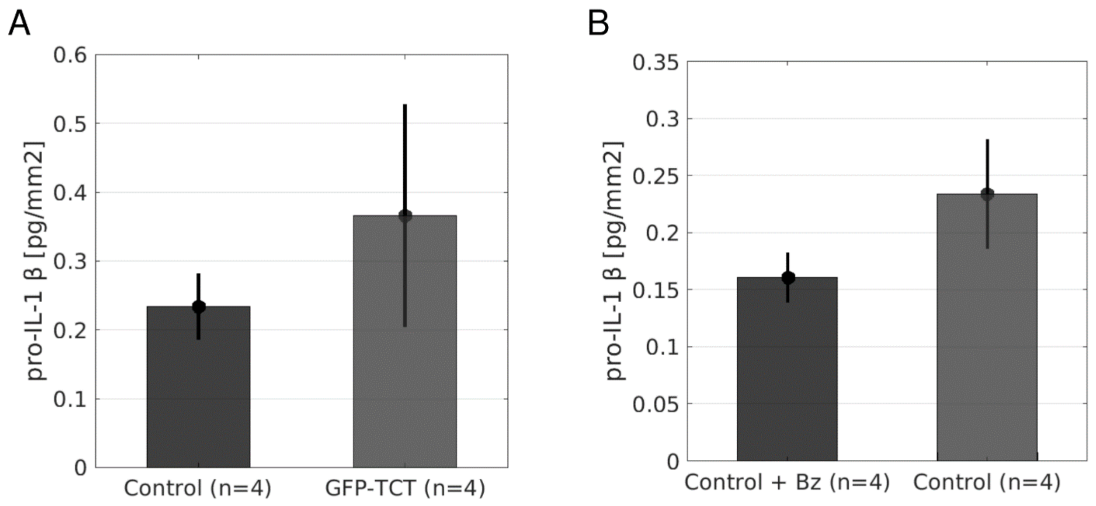

S5 Fig. **Cytokine expression levels in the parasitized HCP.** Hamsters were inoculated with GFP-TCTs and twenty-four hours later, the animals (n = 4) were either treated orally or not (n = 4) with Bz (100 mg/kg/day). **(A)** ELISA measurements of pro-IL-1β detection (pg/mm^2) in the parasitized tissues (6 dpi) as compared to the contralateral HCP (p = 0.1461). **(B)** Pro-IL-1β detection (pg/mm^2) in Bz (>24 h) treated contralateral HCP (7 dpi) as compared to untreated controls (p = 0.0165).

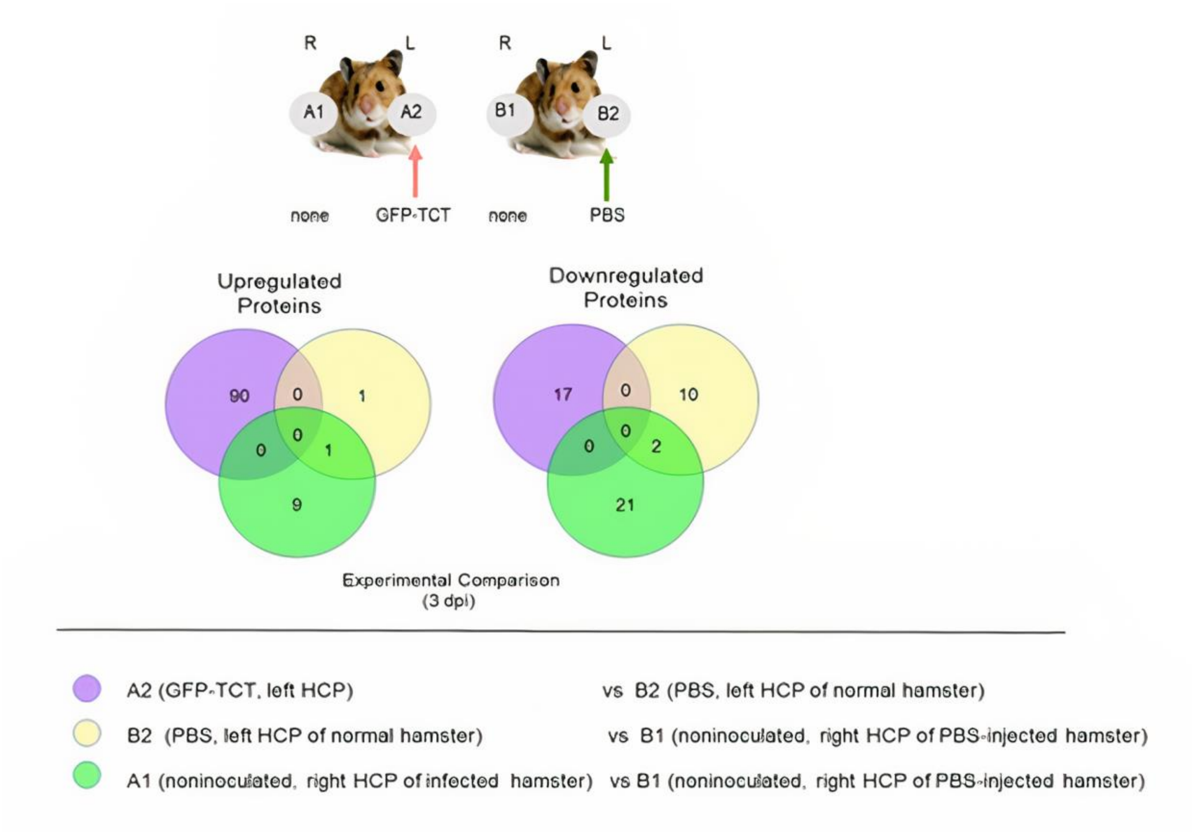

S6 Fig. Venn diagram of proteomic analysis. Cheek pouches (n=2) excised from separate groups of hamsters were inoculated (left HCP) with 10^6 GFP-TCT, and the contralateral pouch was left untouched. Controls involved inoculation of normal hamsters with PBS (left) and the contralateral HCP was left untouched. Animals were euthanized at 3 dpi and the excised HCPs were processed for protein extraction. Proteins were digested with trypsin and peptides labeled with an isobaric tag for absolute and relative quantitation (iTRAQ). Venn diagram depicts upregulated **(A)** and downregulated proteins **(B),** differentially expressed in comparisons, as indicated by letters.

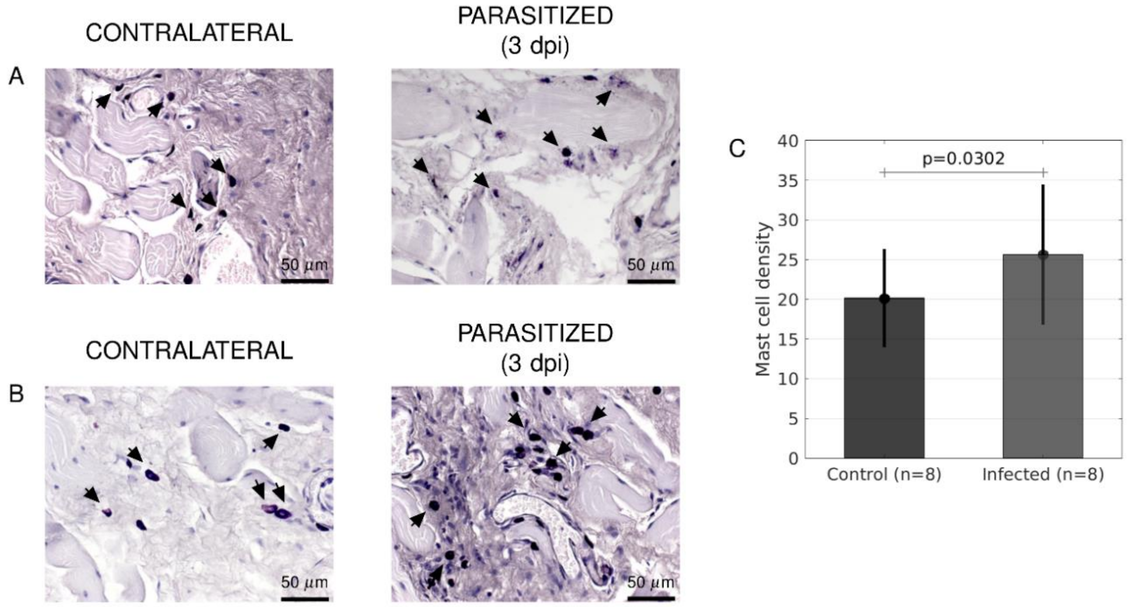

S7 Fig. Mast cell density in parasitized HCP. Toluidine blue staining of HCP tissues injected 3 days earlier with PBS (contralateral) versus 106 GFP-TC (right). Longitudinal sections of 5 µm were photographed in a light microscope and the total number of MCs (black arrows) was determined. **(A)** Staining of contralateral tissues injected with PBS versus infected HCP. Presence of degranulating MCs in parasitized tissues is indicated by arrows. **(B)** MC density scores represent the mean number of MCs per field in control and parasitized tissues, as indicated. **(C)** Means were compared by unilateral paired *t*-test ($p = 0.0302$).

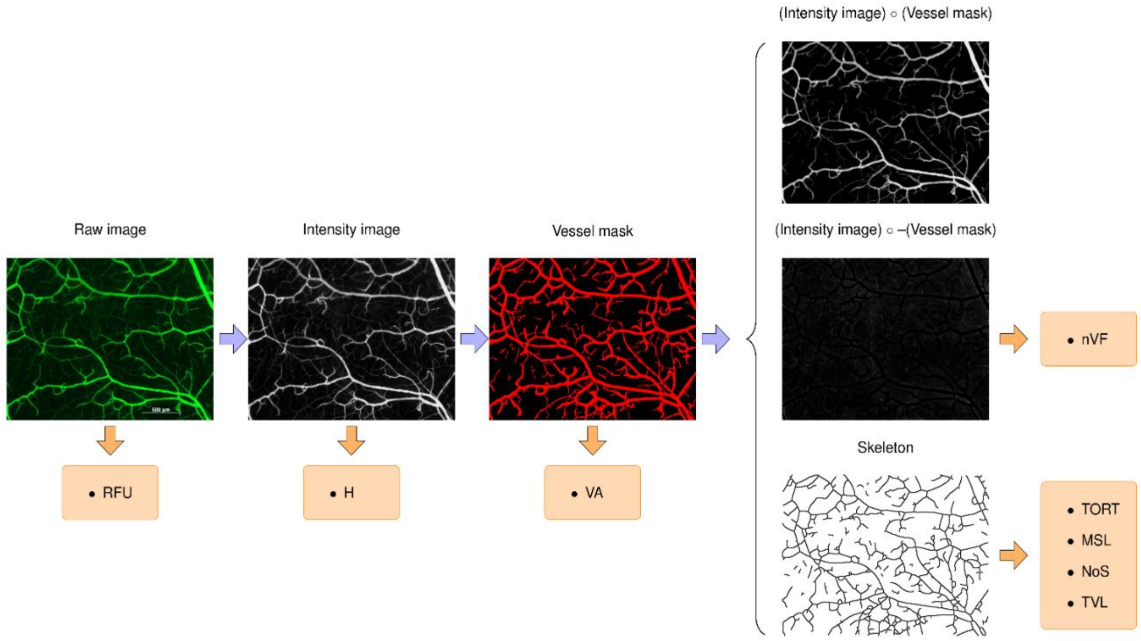

S8 Fig. Image processing methodology: Pipeline features the sequence of steps to move from the Raw image (computation of RFU) to the Intensity image (computation of H), Vessel mask (computation of VA), and then Boolean operations which enable the computation of nVF, TVL, MSL, NoS, and TORT microvascular indexes. Specifically, indexes TVL, MSL, NoS, and TORT are computed using the skeleton that characterizes the topology of the microvasculature.
